# A Genetic Screen Identifies a Requirement for Cysteine-Rich–Receptor-Like Kinases in Rice NH1 (OsNPR1)-Mediated Immunity

**DOI:** 10.1371/journal.pgen.1006049

**Published:** 2016-05-13

**Authors:** Mawsheng Chern, Qiufang Xu, Rebecca S. Bart, Wei Bai, Deling Ruan, Wing Hoi Sze-To, Patrick E. Canlas, Rashmi Jain, Xuewei Chen, Pamela C. Ronald

**Affiliations:** 1 Department of Plant Pathology and the Genome Center, University of California, Davis, Davis, California, United States of America; 2 Joint Bioenergy Institute, Emeryville, California, United States of America; 3 Institute of Plant Protection, Jiangsu Academy of Agricultural Sciences, Nanjing, China; 4 College of Life Sciences, Inner Mongolia Agricultural University, Huhhot, China; 5 Rice Research Institute, Sichuan Agricultural University at Chengdu, Wenjiang, Chengdu, Sichuan, China; Ohio State University, UNITED STATES

## Abstract

Systemic acquired resistance, mediated by the Arabidopsis *NPR1* gene and the rice *NH1* gene, confers broad-spectrum immunity to diverse pathogens. NPR1 and NH1 interact with TGA transcription factors to activate downstream defense genes. Despite the importance of this defense response, the signaling components downstream of NPR1/NH1 and TGA proteins are poorly defined. Here we report the identification of a rice mutant, *snim1*, which suppresses NH1-mediated immunity and demonstrate that two genes encoding previously uncharacterized cysteine-rich-receptor-like kinases (*CRK6* and *CRK10*), complement the *snim1* mutant phenotype. Silencing of *CRK6* and *CRK10* genes individually in the parental genetic background recreates the *snim1* phenotype. We identified a rice mutant in the Kitaake genetic background with a frameshift mutation in *crk10*; this mutant also displays a compromised immune response highlighting the important role of *crk10*. We also show that elevated levels of *NH1* expression lead to enhanced *CRK10* expression and that the rice TGA2.1 protein binds to the *CRK10* promoter. These experiments demonstrate a requirement for CRKs in NH1-mediated immunity and establish a molecular link between NH1 and induction of *CRK10* expression.

## Author Summary

To survive, plants and animals must resist microbial infection. Plants employ an immune response called systemic acquired resistance that confers long-lasting resistance to a broad-spectrum of pathogens. Researchers have previously identified two key proteins (NPR1/NH1 and TGA) that control this immune response. Despite these advances, there remain many gaps in our knowledge and understanding of this important immune response. We have identified a new gene (*CRK10*) required for this immune response; without it, plants are more susceptible to infection. These findings advance basic knowledge of systemic acquired resistance and open the door to a new avenue of research on this exciting and important resistance mechanism.

## Introduction

Despite the lack of circulating immune cells, plants share similarities with animals in their defense responses against pathogens; they both use innate immune systems to counter attacks. For example, plants and animals utilize membrane-localized receptors that detect conserved molecular patterns derived from microbes [1], including peptidoglycan, flagellin, chitin, and sulfated peptides. Another component of the immune system consists of cytoplasmic nucleotide-binding-domain, leucine-rich repeat receptors (NLR) [2] that recognize cognate effector proteins secreted by the microbe into the cell. Plants have a third type of defense system, absent in animals, called systemic acquired resistance (SAR) [3,4]. Activation of this system leads to a long lasting, broad-spectrum defense response.

SAR is induced by the plant hormone salicylic acid (SA), and its analogues 2,6-dichloroisonicotinic acid (INA), probenazole, and benzothiadiazole (BTH) [5–10]. In Arabidopsis, NPR1 (nonexpressor of pathogenesis-related genes 1; also known as *NIM1* and *SAI1*) is the key regulator of SAR [11–15]; SA induces NPR1 expression and activates the NPR1 protein leading to immunity against diverse pathogens [16–19]. NPR1 interacts with TGA transcription factors, which are required for the SAR response [20–24]. NPR1 contains an ankyrin-repeat domain, a BTB/POZ domain, and a C-terminal transcription activation domain. The ankyrin-repeat domain interacts with TGA proteins [20]; the BTB/POZ domain regulates the C-terminal activation domain [25,26]. SA binds NPR1 and may regulate NPR1 directly [27]; another model suggests that SA regulates NPR1 indirectly through NPR3 and NPR4, which bind SA with high affinities [28].

Arabidopsis NIMIN (NIM1 interacting) and rice NRR (negative regulator of resistance) family proteins are the second class of proteins that interact with Arabidopsis NPR1/NIM1 and rice NH1 (NPR1 homolog 1; also known as OsNPR1) [29,30], respectively. Arabidopsis NIMINs [31] and rice NRR members (NRR, RH1, RH2, and RH3) [32] negatively regulate NPR1 and NH1, respectively [31–33].

In rice, elevated expression of Arabidopsis *NPR1* or the rice ortholog *NH1* or the NH1 paralog *NH3* [22,29,30,34] results in enhanced resistance to *Xanthomonas oryzae* pv. *oryzae* (*Xoo*) and *Magnaporthe oryzae*, the causal agents of rice bacterial leaf blight and rice blast, respectively. Like Arabidopsis NPR1, rice NH1 also interacts with TGA transcription factors [29]. The enhanced disease resistance of NH1 overexpression (NH1ox) rice plants is accompanied by and correlated with a spontaneous cell death phenotype, commonly referred to as a lesion mimic phenotype [29,35,36]. Application of BTH enhances the formation of necrotic spots from the spontaneous cell death on the NH1ox plants [29], indicating a tight association between this lesion mimic phenotype and enhanced resistance to *Xoo*.

Although Arabidopsis NPR1 and rice NH1 bind to TGA proteins and act as transcriptional co-activators [26,37], the downstream components required for the NPR1/NH1-mediated response remain largely uncharacterized. To identify such proteins, we screened a fast-neutron-induced rice mutant population of NH1ox plants treated with BTH and identified a mutant called *s**uppressor of* *N**H1-mediated* *im**munity 1* (*snim1*) that no longer responds to BTH. Here, we report the identification of two previously uncharacterized cysteine-rich receptor-like kinases (encoded by *CRK6* and *CRK10*) that complement the *snim1* phenotype and are required for the BTH-induced immune response. We demonstrate that elevated *NH1* levels induce *CRK10* expression and that the rice TGA2.1 protein binds to the *CRK10* promoter, indicating that both NH1 and TGA proteins regulate *CRK10* expression.

## Results

### Identification of the *snim1* mutant and an 88-kb deletion associated with the suppressor phenotype

**Fig 1:**
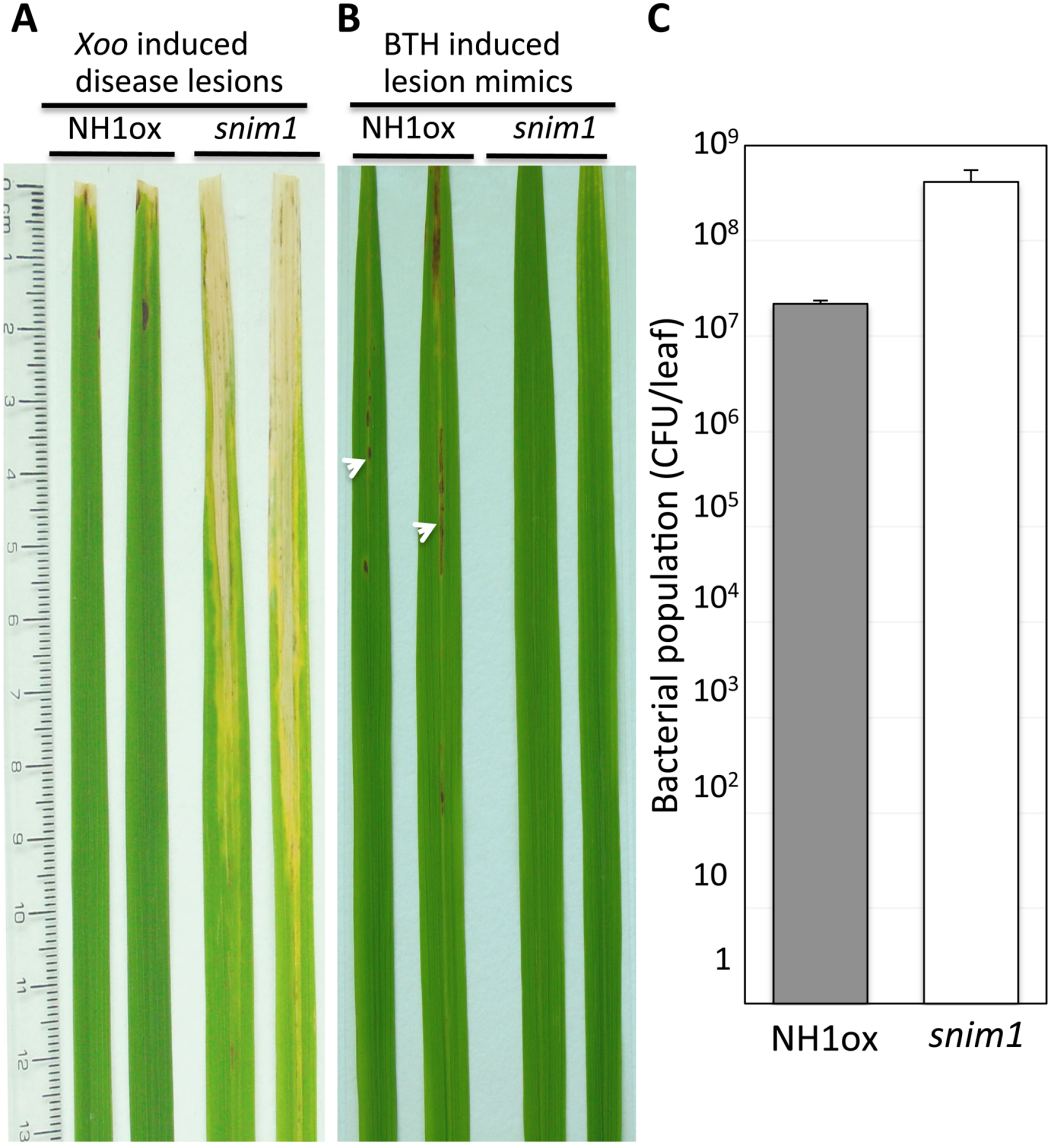
The *snim1* mutant is compromised in immunity to *Xoo* and BTH-induced necrotic lesion formation. Two representative leaves are displayed for the NH1ox parent and the *snim1* mutant in (A) and (B). Inoculation with *Xoo* was carried out with the scissor-dip method (see Methods). (A) *Xoo*-induced, long water-soaked disease lesions in the *snim1* mutant. (B) Lesion mimic necrotic spots. The necrotic spots (marked with arrowheads) developed one week after application of 1 mM BTH. (C) Bacterial populations determined 14 days after inoculation. T-test yielded P = 0.0052. Each bar represents the mean and standard deviation of three leaves.

In our screen for suppressors of NH1-mediated immunity [38], we isolated the *snim1* mutant. After inoculation with *Xanthomonas oryzae* pv. *oryzae* (*Xoo*), *snim1* develops long water-soaked lesions, characteristic of the disease. In contrast, the parental control NH1ox plants are resistant to infection, resulting in short lesions (Fig 1A). Furthermore, the NH1ox parent displays a lesion mimic spontaneous cell death phenotype after application of BTH, which is a plant defense activator [38], but *snim1* lacks this phenotype (Fig 1B). Bacterial population measurements show that *snim1* harbors 18 times more *Xoo* than NH1ox plants (P = 0.0052) (Fig 1C). These results indicate that the *snim1* mutation impairs NH1-mediated immunity.

**Fig 2:**
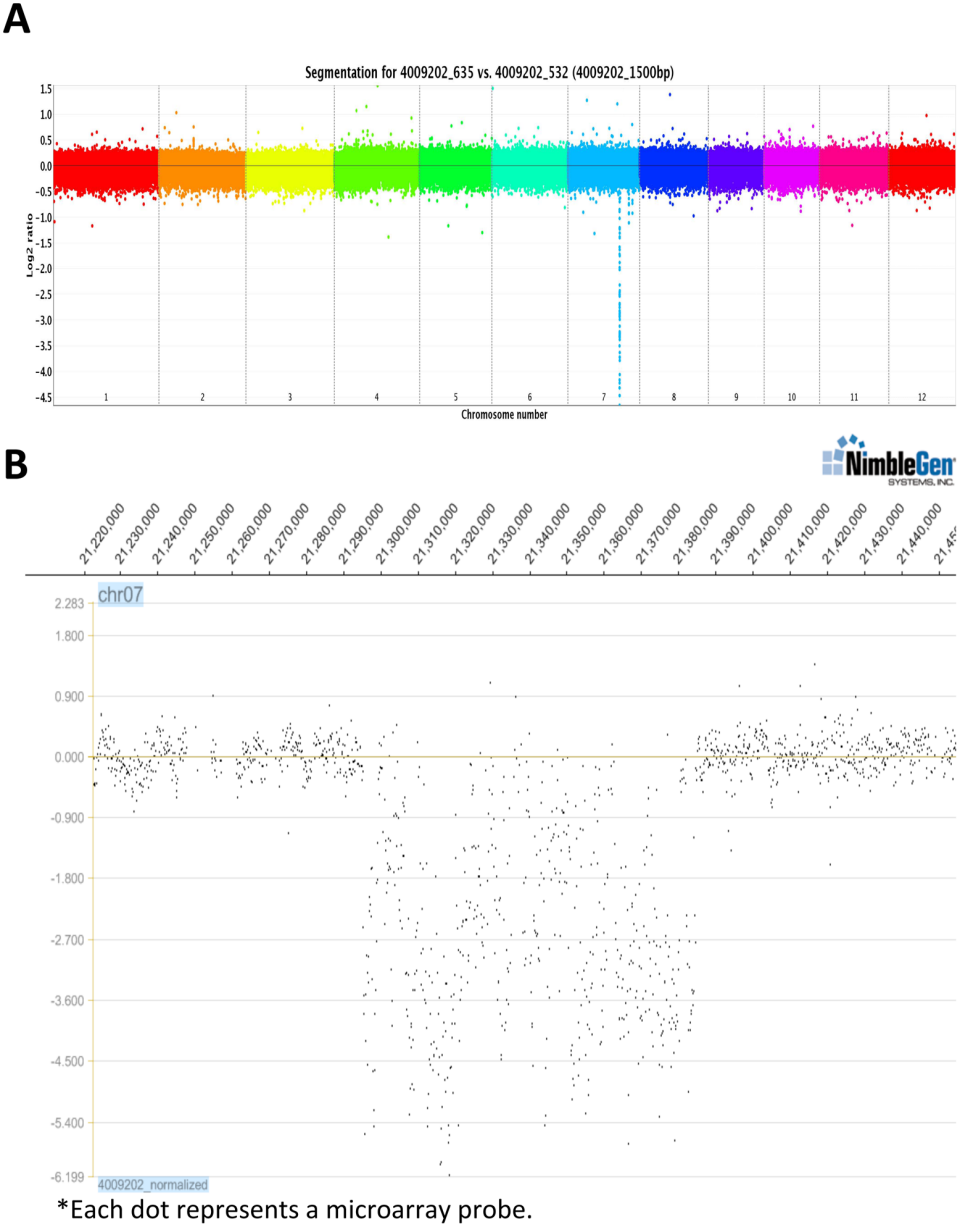
Comparative genome hybridization identifies a deletion in *snim1*. (A) A composite CGH graph. Each color represents a chromosome; the downward peak represents a deletion. The codes 4009202–635 vs 532 represent *snim1* vs NH1ox DNA samples. Each dot represents a segment of 1500 bp. The Y-axis is in log2 scale. (B) An enlarged view of part of chromosome 7 that contains the deleted region. Expression level is in log2 scale. Each dot represents an actual microarray probe.

To expedite isolation of the gene(s) responsible for the *snim1* phenotype, we conducted comparative genome hybridization (CGH) analysis using a NimbleGen 2.1-million-probe rice tiling array [38] comparing *snim1* and NH1ox DNA (Fig 2A). These experiments identified a single large deletion (from approximately 21,297,000 to 21,385,000) on chromosome 7 of *snim1* (Fig 2B). This 88kb deletion was confirmed by PCR. The deleted region contains 11 genes: six *CRKs*, one encoding development and cell death-kelch motif protein, three encoding expressed proteins, and one Ty3-gypsy retrotransposon gene. Their MSU gene IDs and relative positions are shown in S1 Fig.

**Fig 3:**
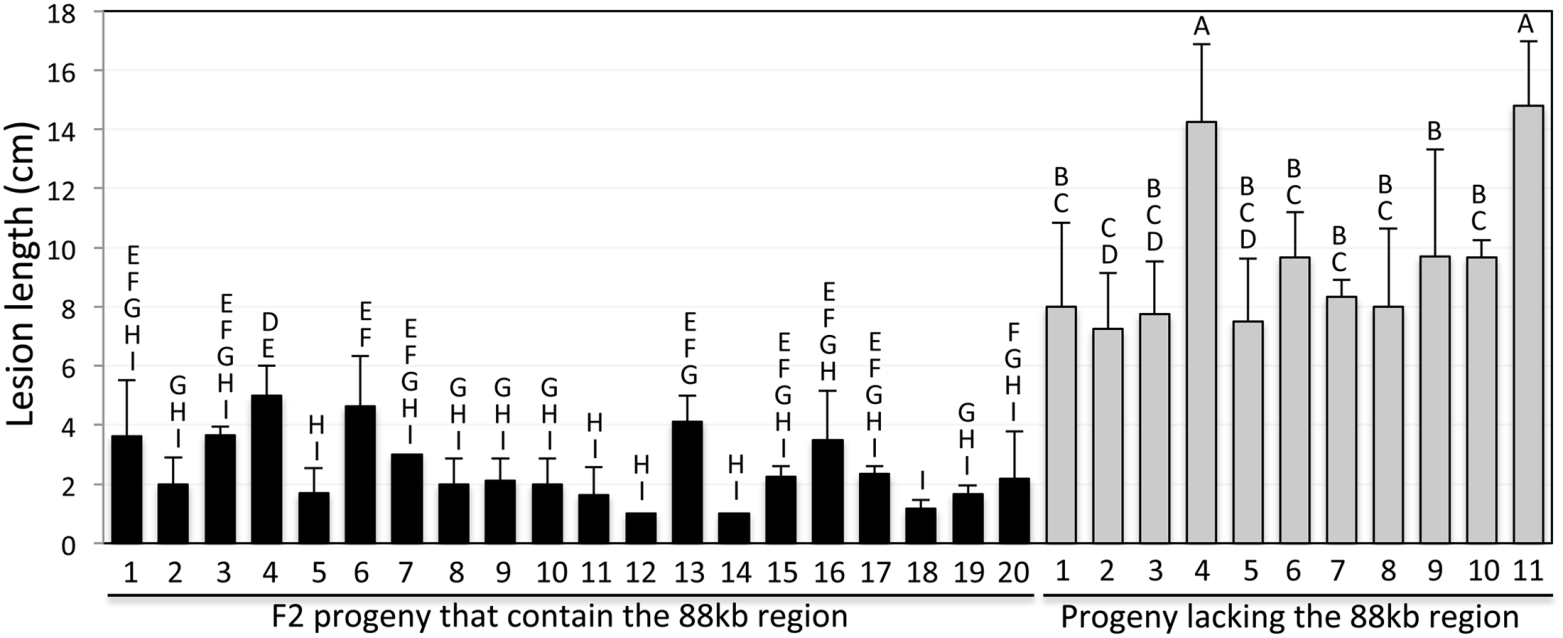
The 88-kb deletion cosegregates with the *snim1* phenotype. A segregating progeny population derived from a cross between the *snim1* mutant and the LG parent was genotyped for the presence of the *Ubi-NH1* gene (NH1ox) and the *CRK10* gene (representing the 88-kb region) and scored for resistance to *Xoo*. Only progeny containing the *Ubi-NH1* gene are shown. *CRK10* positive and negative progeny plants are separated into two groups. The letters above each bar show the statistical groupings using the student T-test on each pair based on the 5% significance level.

Analysis of F2 progeny derived from a cross between *snim1* and the parent reveals a complete association of the 88kb deletion with the *snim1* phenotype (Fig 3). F2 progeny that contain the 88kb region exhibit levels of *Xoo* resistance similar to that of the NH1ox parent (Fig 3). In contrast, F2 progeny lacking the 88kb region are susceptible to *Xoo*. T-test yields P<0.0001, indicating the difference between the two groups is highly significant.

### *CRK6* and *CRK10* complement the *snim1* phenotype and silencing of *CRK6* and *CRK10* individually compromises NH1-mediated immunity

**Fig 4:**
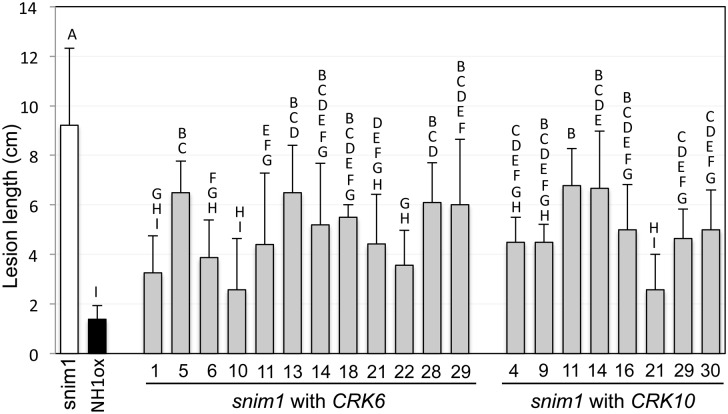
*CRK6* and *CRK10* complement *snim1*. The *snim1* complementation lines containing either *CRK6* or *CRK10* were inoculated with *Xoo*. Each bar represents the average and standard deviation of at least three leaves from an independent transgenic line. Letters show statistical groupings (P<0.05).

To assess the involvement of the 10 non-retrotransposon genes deleted in *snim1*, we conducted complementation experiments. We ligated each isolated gene into the binary vector C4300, and used the resulting construct to transform *snim1*. Following inoculation with *Xoo*, we found that only the *CRK6* and *CRK10* constructs restore resistance to the *snim1* mutant (Fig 4). None of the other 8 genes restore resistance to *Xoo* (S2 Fig).

To investigate the relationship between the rice CRKs and their Arabidopsis paralogs, we constructed a phylogenetic tree for the 45 CRKs and included the Arabidopsis CRKs to display their relative positions (S3 Fig).

**Fig 5:**
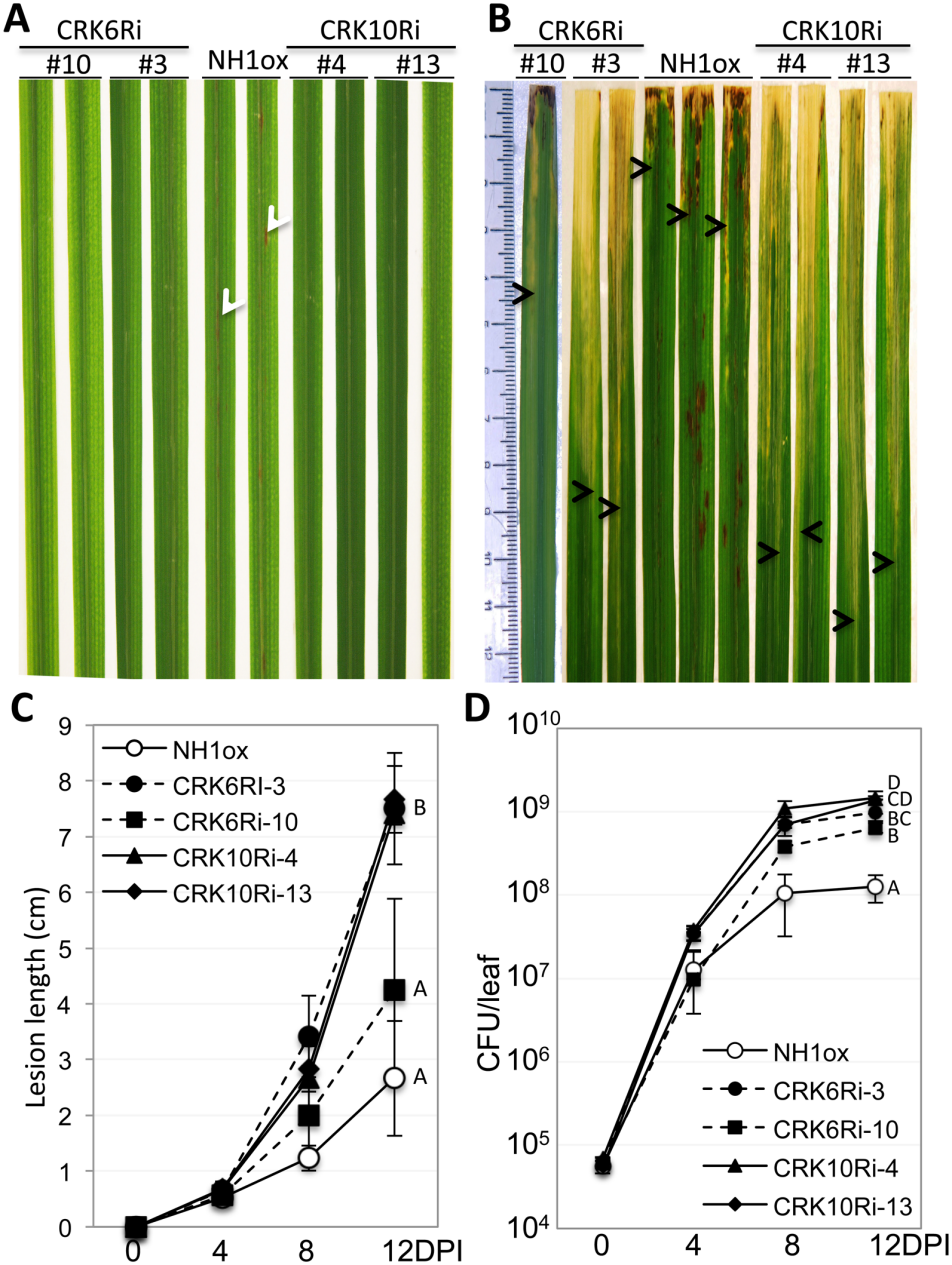
Silencing of *CRK6* or *CRK10* re-creates the *snim1* phenotypes. CRK6Ri and CRK10Ri plants were treated with 1 mM BTH before *Xoo* inoculation. Two lines each of CRK6Ri and CRK10Ri are shown. (A) Lesion mimic formation after BTH treatment. Lesion mimic spots on NH1ox leaves are indicated by white arrowheads. (B) *Xoo*-induced water-soaked lesions. The bottom boundary of the lesion on each leaf is marked with an arrowhead to indicate the lesion lengths. Leaves were taken 14 days post inoculation. (C) Lesion length development after *Xoo* inoculation. Each time point contains 6 leaves. (D) Bacterial growth curves. Each time point represents three replicates. Statistical groupings: A = CRK10Ri-4, AB = CRK10Ri-13, BC = CRK6Ri-3, C = CRK6Ri-10, D = NH1ox.

To further validate the requirement of *CRK6* and *CRK10* for NH1-mediated immunity to *Xoo*, we silenced *CRK6* and *CRK10* individually in the NH1ox background using RNA interference (Ri) and inoculated the resulting six transgenic lines with *Xoo*. Real-time quantitative reverse transcription (qRT)-PCR experiments revealed that the CRK6Ri and CRK10Ri lines are effectively silenced for *CRK6* and *CRK10* expression (70%-90% reduction) (S4 Fig). Accumulation of *CRK6* and *CRK g35580* RNAs are also reduced in the CRK10Ri lines. In contrast, RNA levels of *CRK g35650*, *g35660* and *g35680* are not reduced in the CRK10Ri lines. These results indicate that silencing of *CRK10* also affects *CRK6* and *CRK g35580* expression levels. Based on the low sequence similarity of CRK6 and CRK g35580 with the region of CRK10 used for silencing, we hypothesize that the observed reduced expression is indirect and not due to co-silencing. In contrast to the resistant NH1ox parent plants, which display spontaneous cell death, lesion mimic spots following BTH treatment (Fig 5A) and are resistant to *Xoo* (Fig 5B), the CRK6Ri and CRK10Ri lines lack the spontaneous cell death phenotype (Fig 5A) and are susceptible to *Xoo* (Fig 5B and 5C). The presence of the *CRK10Ri* and *CRK6Ri* transgenes cosegregates with susceptibility to *Xoo* in T1 progeny (S5 and S6 Figs). Bacterial growth curve analyses reveal that CRK6Ri (lines #3&10) and CRK10Ri (#4&13) plants harbor 5–8 times and 11–12 times more *Xoo* than the NH1ox parent, respectively (Fig 5D). Statistical analysis of bacterial populations at day 12 (Fig 5D) indicated significant differences between NH1ox parent, CRK6Ri, and CRK10Ri lines. These results confirm the requirement of *CRK6* and *CRK10* for NH1-mediated immunity.

### The *snim1* and *CRK1*0 mutations compromise BTH-induced resistance to *Xoo* independent of ectopic NH1 over-expression

**Fig 6:**
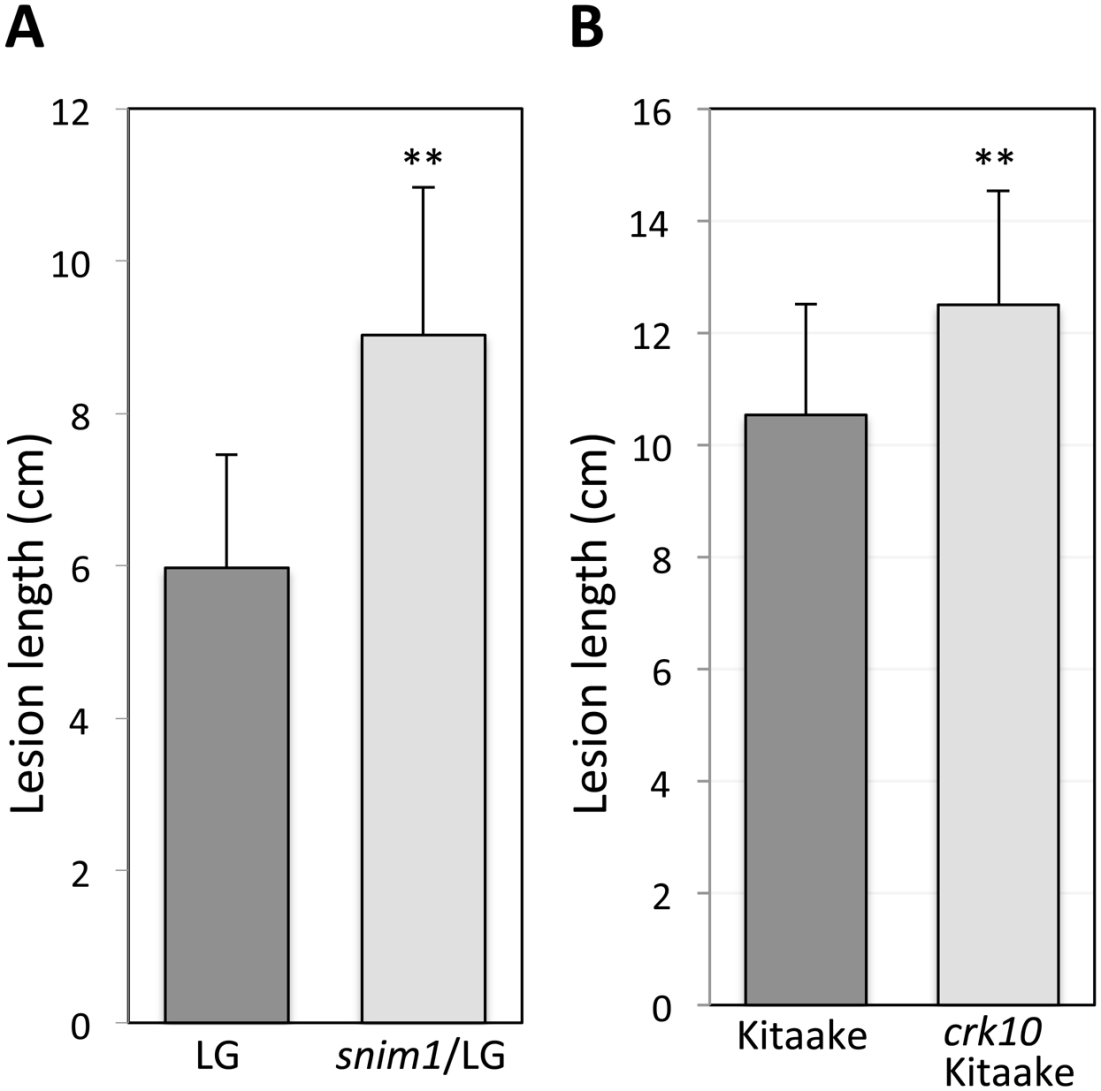
The *snim1* and *crk10* mutations affect resistance to *Xoo* in the wild type genetic background. (A) A population of 27 plants (93 leaves) of the wild type Liaogeng (LG) control and 27 plants (110 leaves) of snim1/LG were inoculated with *Xoo*. Lesion lengths were measured 14 days after inoculation. The average lesion lengths and standard deviations are shown. T-test between LG and snim1/LG gave a P value lower than 0.0001. (B) A population of 27 plants (147 leaves) of *crk10*/Kitaake (FN892-S) was inoculated with *Xoo* together with 25 plants (136 leaves) of the Kitaake control and lesions measured 14 days post inoculation. T-test gave a P = 0.0009.

We next tested whether the *snim1* mutation affects resistance to *Xoo* in the absence of the *NH1ox* transgene. For this purpose, we crossed *snim1* with the parental rice cultivar LiaoGeng (LG), which lacks the *NH1ox* transgene. We identified 27 individual progeny (*snim1*/LG) from this cross that carry homozygous *snim1* 88kb deletion and lack the *NH1ox* transgene. We pretreated the *snim1*/LG progeny and the LG control with 1mM BTH and then inoculated with *Xoo*. The *snim1*/LG individuals display significantly (P<0.0001) longer lesions (9.0±1.9 cm) than the LG control (6.0±1.5 cm) (Fig 6A). These results indicate that the *snim1* mutation also affects BTH-induced resistance to *Xoo* in the absence of the *NH1ox* transgene.

We further verified the role of the *CRK10* gene with a *crk10* rice mutant. We identified a homozygous *crk10* mutant from a fast neutron mutagenized rice population in the Kitaake genetic background [39]. This mutant carries a two-base deletion (S7 Fig) in the third exon of *CRK10* causing a frameshift, resulting in a predicted truncated CRK10 protein missing the kinase domain (see Methods). To assess the effect of the CRK10 mutation in this second genetic background, we inoculated 27 progeny plants of this *crk10* mutant along with 25 wild type Kitaake control plants with *Xoo* following 1mM BTH treatment. We observed that the *crk10*/Kitaake mutant plants develop significantly (P = 0.0009) longer lesions (~12.5 cm) than the control Kitaake plants (~10.5 cm) (Fig 6B). These results further support the conclusion that the *CRK10* gene is required for resistance to *Xoo*.

### Elevated expression of *CRK10* enhances resistance to *Xoo*

**Fig 7:**
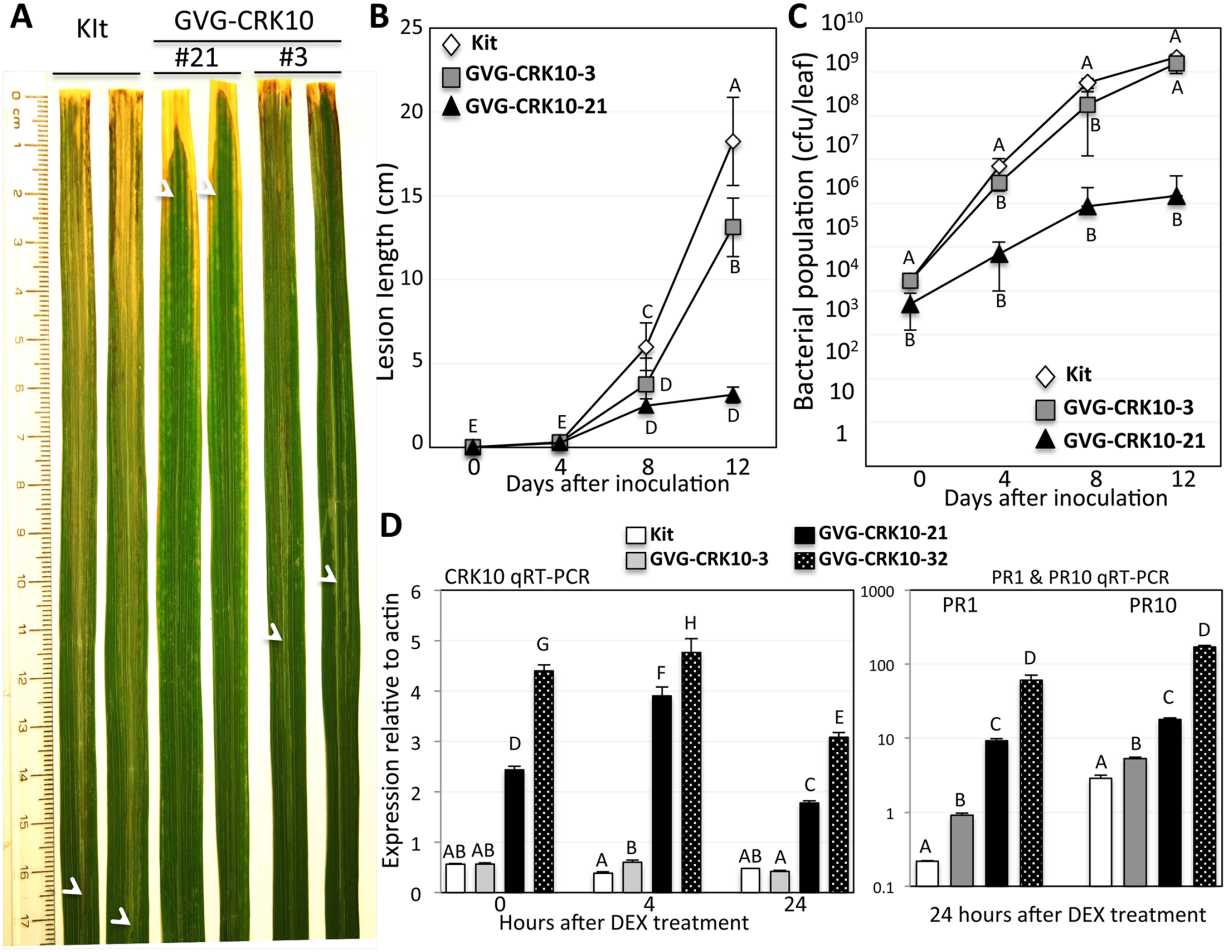
Inducible overexpression of *CRK10* enhances resistance to *Xoo*. (A) Two leaves each of GVG-CRK10 lines (#3 and #21) and the Kitaake control were photographed 2 weeks after *Xoo* inoculation. The lower limit of the *Xoo*-induced water soaked lesions on each leaf is marked with a white arrowhead. (B) Lesion length development and (C) bacterial growth curves were determined over 12 days after *Xoo* inoculation. Each time point represents the average and standard deviation of four leaves. The letter next to each time point represents the statistical grouping based on the 5% significance level. (D) RNA levels of *CRK10* and *PR* genes in GVG-CRK10 lines and the Kitaake control. Real time RT-PCRs for *CRK10* were carried out with RNA extracted from leaves collected before DEX application (0 hours) and at 4 and 24 hours after 100 μM DEX application. *PR1* and *PR10* RNA levels were assessed in the samples treated with DEX for 24 hours. The letter above each bar represents the statistical grouping based on the 5% significance level.

To test the hypothesis that higher levels of *CRK10* enhance resistance, we generated an inducible construct (GVG-CRK10) using the GVG-dexamethasone (DEX) inducible system [40,41] and obtained 10 healthy, independently transformed lines. These plants display enhanced resistance to *Xoo* after DEX induction (Fig 7A–7C). The enhanced resistance cosegregates with the *GVG-CRK10* transgene in T1 segregating progeny (S8 Fig). Further analysis indicates that higher levels of *CRK10* lead to higher levels of resistance to *Xoo* (Fig 7D). Overexpression of *CRK6* did not confer enhanced resistance to *Xoo* (S9 Fig).

### Expression levels of *CRK10* are dependent on NH1 levels and BTH induction

**Fig 8:**
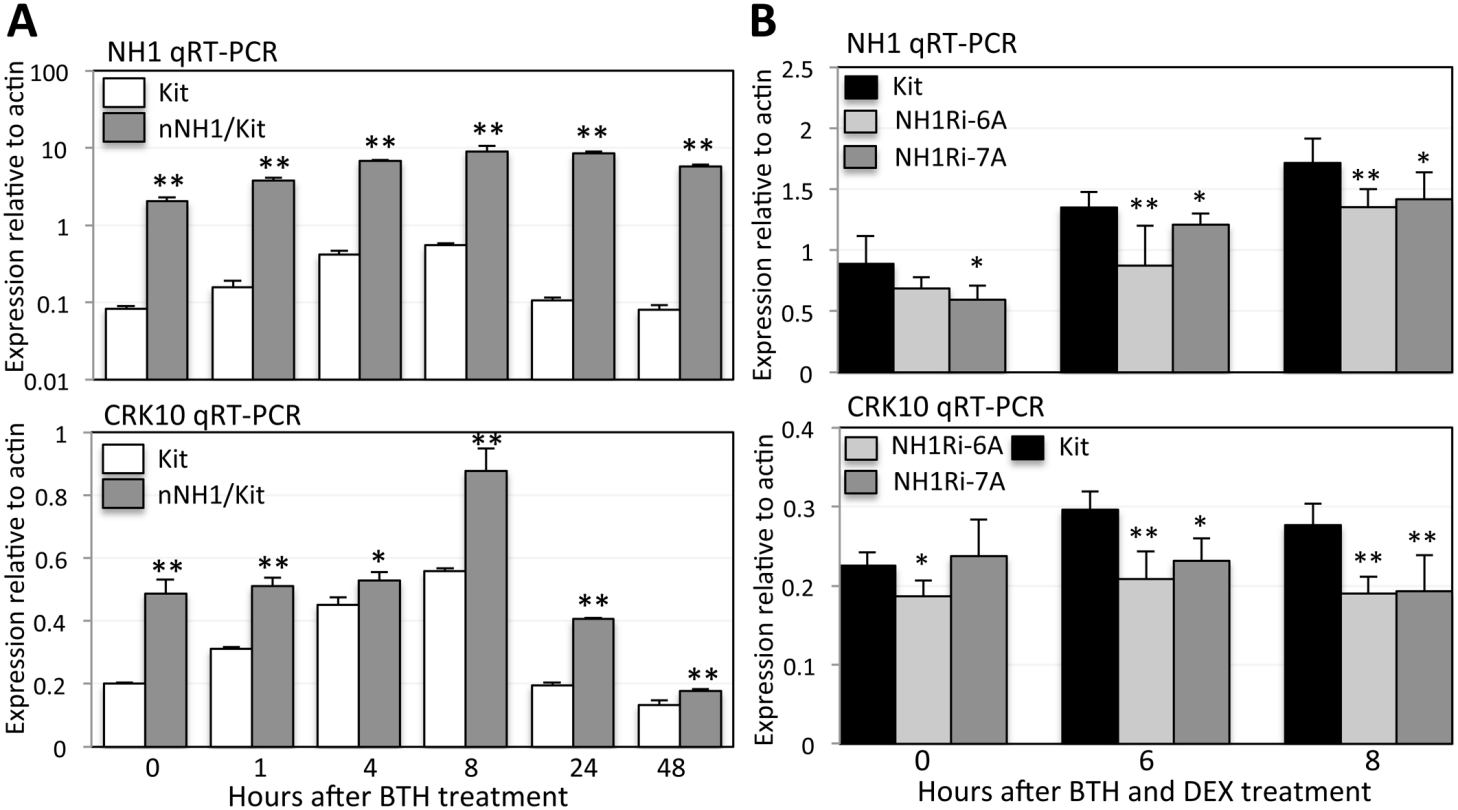
NH1 regulates *CRK10* expression. (A) BTH and NH1 induce *CRK10* expression. Kitaake (Kit) and nNH1 plants, which contain an extra copy of *NH1* and overexpress NH1, were treated with 1mM BTH and leaf samples taken at time points 1, 4, 8, 24, and 48 hours after treatment. The nNH1 samples were compared to the Kit control at each time point for statistical analysis. (B) NH1-silencing reduces *CRK10* expression. Leaves were treated with 1 mM BTH and 100 μM DEX. Each sample represents two biological and three technical replicates. The NH1Ri samples were compared to the Kit control at each time point for statistical analysis. One * indicates P< 0.05 and two indicates P<0.01.

To determine if NH1 regulates expression of *CRK* genes, we assessed *CRK10* and *CRK6* expression levels in Kitaake and NH1 overexpression plants (nNH1 in Kitaake genetic background). We treated plants with 1 mM BTH and collected leaf samples 0, 1, 4, 8, 24, and 48 hours after treatment. BTH treatment induces *NH1* expression levels in both Kitaake (labeled Kit) and nNH1 plants, reaching peaks at 8 hours (Fig 8A). BTH treatment induces 3-fold higher levels of *CRK10* mRNA (at 8 hr) in Kitaake compared with the untreated control (0 hr); this induction is higher (4.5-fold) in the nNH1 plants (at 8 hr; Fig 8A). In the absence of BTH pretreatment, the *CRK10* mRNA level is 2.5-fold higher in nNH1 plants compared with control plants. BTH treatment also induces *CRK6* expression (by 4 fold at 8hr). We observed a slight delay in *CRK6* induction in nNH1 plants. This delay does not significantly change the basal and peak levels of *CRK6* expression (S10 Fig).

To further investigate the observation that NH1 regulates *CRK10* expression, we assessed *CRK10* expression levels in transgenic Kitaake plants carrying an NH1 RNAi construct (NH1Ri), under control of the DEX-inducible promoter. The *NH1* mRNA levels are significantly reduced in the NH1Ri plants (NH1Ri-6A and -7A) silenced for expression of NH1 compared with the Kitaake control, 6 and 8 hours after DEX and BTH treatment (Fig 8B). Importantly, we also observed that *CRK10* mRNA levels are also significantly reduced in the NH1Ri plants compared with the Kitaake control (Fig 8B). These results indicate that BTH and *NH1* regulate *CRK10* expression.

### The *CRK10* promoter contains a TGA binding site

**Fig 9:**
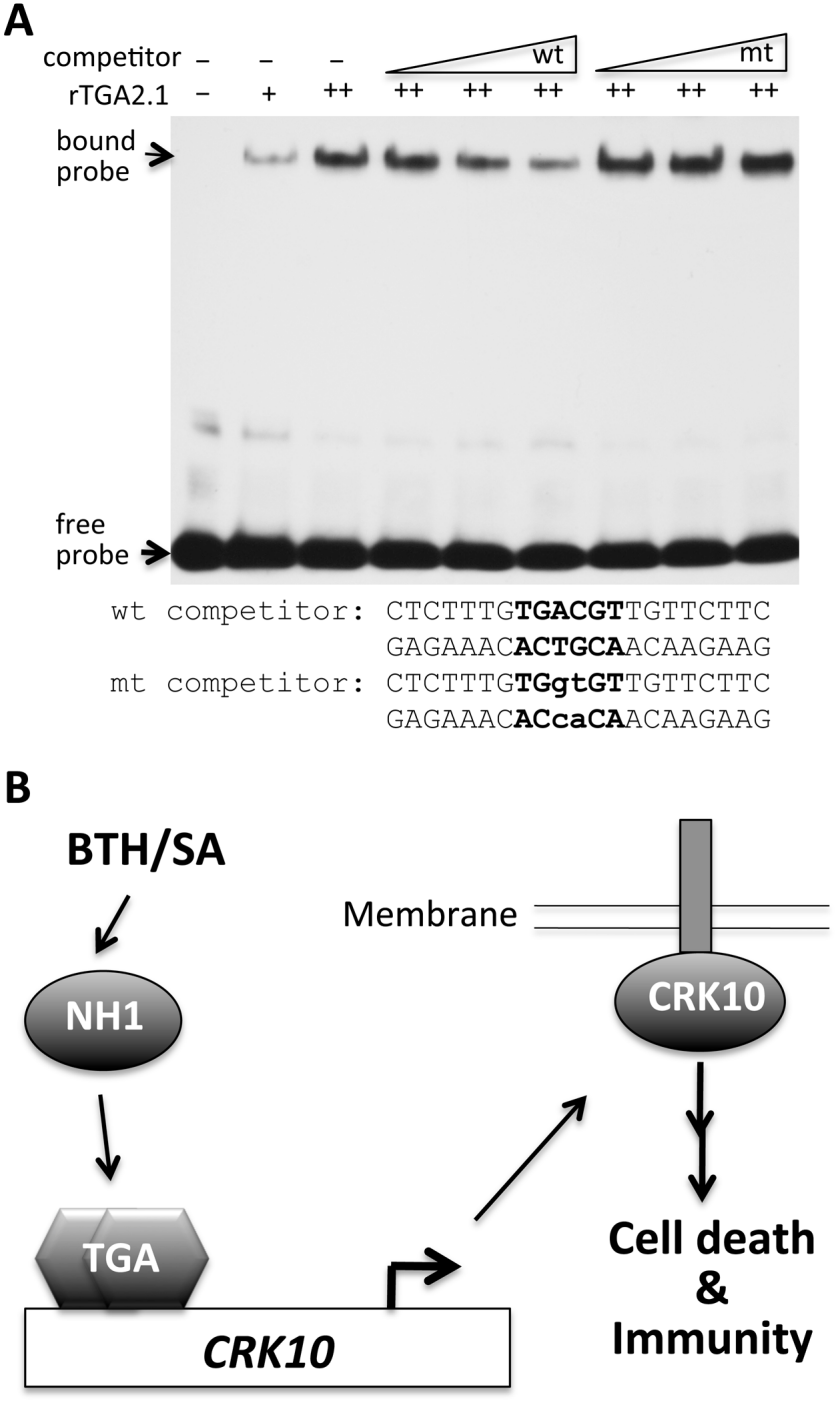
Binding of a rice TGA protein to the *CRK10* promoter and a summary model. (A) Rice TGA2.1 binds to the *CRK10* promoter in a gel mobility shift assay. 5X, 20X, 100X of unlabeled wild-type (wt) and mutated (mt) oligonucleotides were included as competitors (sequences as shown). The probe contains the same sequence as the wt competitor, but is coupled to a biotin at the 5’-end. (B) A model for NH1, TGA, and CRK10-mediated immunity.

To assess the mechanism by which *CRK10* expression is activated, we examined the *CRK10* promoter for potential transcription factor binding sites. We identified the sequences TGACGT (-793 from the TATA box) and TGACG (-164) in the *CRK10* and *CRK6* promoters, respectively, that match the consensus sequence binding sites for TGA transcription factors. We synthesized an oligonucleotide containing this putative *CRK10* TGA binding site and employed an electrophoresis mobility shift assay (EMSA) to test its interaction with the rice TGA2.1 protein, which has been shown to bind to an SA-responsive element and interact with NH1 [42,43]. We found that rice TGA2.1 binds to the synthetic oligonucleotide and that the observed gel shift can be competed with higher concentrations of wild-type oligonucleotides but not by oligonucleotides carrying a mutation in the TGA binding site (Fig 9A). These results demonstrate that the *CRK10* promoter contains a cognate TGA binding site.

### CRK6 is an active kinase

CRK6 and CRK10 carry conserved kinase motifs. To assess the kinase activity of CRK6, we fused the CRK6 kinase domain (CRK6K) to the His:Nus protein, expressed the fusion construct in *E*. *coli* and purified the fusion protein using Ni-NTA resins. As a negative control, we generated a kinase-dead mutant of the CRK6 kinase by mutating the conserved, required aspartate at amino acid 488 to asparagine (CRK6DN). The two fusion proteins were subject to kinase activity assay (see Methods). The Nus:CRK6K protein shows clear autophosphorylation, whereas the Nus:CRK6DN protein did not (S11A Fig). These results indicate that CRK6 is an active kinase. We were unable to express and purify the CRK10 kinase. However, the kinase domains of CRK6 and CRK10 are highly conserved sharing 76% similarity (S11B Fig), including all amino acids known to be critical for kinase activity suggesting that CRK10 is likely also an active kinase.

In addition to the kinase domain, we have also analyzed the CRK6 and CRK10 proteins for other conserved protein domains with the SMART program, which is specialized in detecting protein domains. Both CRK6 (amino acids 1–31) and CRK10 (aa. 1–27) contain a predicted signal peptide. Both also contain a predicted transmembrane region: amino acids 295–317 for CRK6 and 284–306 for CRK10. These results strongly predict that CRK6 and CRK10 are membrane-localized proteins.

## Discussion

In this manuscript we demonstrate that the previously uncharacterized proteins, CRK6 and CRK10, are required for BTH-inducible, NH1-mediated immunity in rice. There are 43 additional CRK genes in rice (http://rice.plantbiology.msu.edu) and at least 44 CRK members in Arabidopsis [44,45]. In both rice and Arabidopsis, many of the *CRK* genes are clustered together in the genome. This structure may facilitate recombination and accelerate evolution of resistance. A similar mechanism for generating diversity has been postulated for plant resistance genes encoding both nucleotide-binding site leucine-rich repeats proteins and leucine rich repeat receptor kinases [46,47].

Although altered expression of *CRK* genes has been observed in several datasets in response to biotic stress [48–53], the biological functions of *CRK* genes have not previously been well elucidated for any of the Arabidopsis or rice *CRK* genes for their involvement in the immune response. In particular, the requirement of these genes in NPR1- or NH1-mediated immunity has not previously been demonstrated. The observed functional redundancy of *CRK* genes in monocots and dicots has likely hindered their characterization and prevented an unambiguous assignment of their function [53]. Because of these complications, previous analyses of CRK proteins relied almost exclusively on overexpression experiments. For example, overexpression of *AtCRK4*, *AtCRK5*, *AtCRK6*, *AtCRK13*, and *AtCRK45* resulted in cell death, activation of defense genes, and/or increased resistance to *Pseudomonas syringae* pv. *tomato* DC3000. Of the knockout analyses conducted, only the *Atcrk45* mutant displayed a slight alteration in response to *Pst* DC3000 [50]. Here we provide direct and robust genetic evidence that the *CRK6* and *CRK10* genes mediate BTH-induced immune response.

We have demonstrated that the *snim1* mutant and the *crk10* knockout mutant compromise resistance to *Xoo* in two genetic backgrounds in the absence of *NH1* overexpression (Fig 6). Furthermore, *CRK10* is likely broadly involved in resistance to pathogens in a quantitative manner because higher levels of *CRK10* correlate with enhanced cell death and immune response (Fig 7). This result is consistent with the observations that *CRK* genes are induced by diverse pathogens [48–53]. *CRK10* may play a more significant role in the immune response than other rice *CRK* genes because our results clearly show that silencing (Fig 5) and knockout (Fig 6B) of *CRK10* itself both cause obvious phenotypes in the immune response; *CRK6*-silenced lines show a less susceptible phenotype.

Our genetic data reveal that elevated expression of NH1 results in enhanced *CRK10* expression. Conversely, a reduction in *NH1* expression leads to a reduction of *CRK10* expression. These results unambiguously demonstrate that *CRK10* expression is regulated by the NH1 protein. The observations that TGA proteins bind to the *CRK10* promoter (Fig 9A) and interact with NH1 [43] suggest that NH1 and TGA proteins function together to activate expression of *CRK10*. Fig 9B presents a model for NH1 and CRK10-mediated activation of defense responses. Based on the observation that NPR1 or NPR3/NPR4 proteins bind SA [27,28], we hypothesize that rice NH1 also senses BTH/SA and that the activated NH1 protein translocates to the nucleus where it interacts with TGA transcription factors. The TGA/NH1 protein complex then activates expression of downstream genes, including *CRK6/CRK10*.

## Methods

### Plant materials, screening, and *Xoo* inoculation

The rice mutant population in the NH1ox-54 genetic background (in the rice LiaoGeng variety carrying the *Ubi-NH1* gene) was generated by irradiation with fast neutrons at 20 Grays as previously described [38]. *Xoo* inoculation was carried out in a growth chamber, set at 26°C with 80% humidity. Inoculation of rice plants with *Xoo* strain PXO99 was carried out with the scissor-dip method [54] with absorbance (600 nm) at OD = 0.5. The *crk10*/Kitaake mutant was generated in the *Xa21* background and was inoculated with the *ΔraxST/PXO99* strain, which evades *Xa21*-mediated immunity [55].

### Comparative genome hybridization

Comparative genome hybridization was carried out at the Roche NimbleGen facility (Madison, WI) using the Roche NimbleGen rice whole genome tiling array as described before [38].

### Cloning of individual genes on chromosome 7 for test to complement the *snim1* mutant

A Qiagen Long Range PCR kit was used for amplification of each gene including the promoter, the coding region, and the 3’ sequence, from chromosome 7. Amplification of g35580 used primers G580-1 and G580-3, g35600 used primers G600-1 and G600-2, g35610 used primers G610-1 and G610-3, g35620 used primers G620-1a and G620-2a, g35630 used primers G630-1 and G630-2, g35640 used primers G640-1 and G640-2, g35650 used primers G650-1 and G650-2, g35660 used primers G660-1 and G660-2, g35680 used primers G680-1 and G680-3, g35690 used primers G690-1 and G690-3, and g35700 used primers G700-1 and G700-2. Amplification of g35600, g35610, g35640, g35650, g35660, g35690, g35700 used Liaogeng genomic DNA as the PCR template. The remaining genes used PAC clone P0458H05 as template. PCR products were cloned into the pCR8/GW/TOPO vector (Invitrogen) and confirmed by sequencing. Each gene was subcloned into the C4300 vector by Gateway recombination.

Genotyping of plants carrying the 88-kb deletion was carried out with primers targeting genes *CRK6*, *CRK10*, or Os07g35610. *CRK6* genotyping used primers G690-RT1 and G690-RT2. *CRK10* genotyping used primers G700-RT3 and G700-RT4. Os07g35610 genotyping used primers G610-10 and G610-2.

### *crk10* mutant in kitaake

A two-base deletion in exon 3 of the *CRK10* gene (Os07g35700) is present (line # FN892-S, M2) compared to the Kitaake parent that causes a frame shift disrupting the *CRK10* open reading frame. M3 progeny plants were used for *Xoo* inoculation.

### Plasmid construction for gene silencing and overexpression

To generate an RNAi construct targeting *CRK6* (Os07g35690), we used primers G690-SiRI and G690-SiBam to amplify a 500bp fragment from the 5’-end of *CRK6*. This fragment was digested with EcoRI and BamHI and cloned into plasmid pENTR/L16, modified from pENTR/D to contain multiple cloning sites. The clone was confirmed by sequencing. The fragment was excised with EcoRI and BamHI and subcloned into pBluescript II SK-, pre-cut with BamHI and phosphatase-treated, jointly with the *Xa21* intron (precut with EcoRI). The resulting clone (dsG690/SK) contained two pieces of the *CRK6* fragment head-to-head with the *Xa21* intron in between to serve as a spacer to stabilize the clone in bacteria. The dsG690 insert was excised with BamHI and subcloned back to the pENTR/L16 vector using the BamHI site and recombined with a Gateway compatible Ubi-C4300 binary vector (Ubi-C4300/GA) to yield construct Ubi-dsG690/C4300. To generate an RNAi construct targeting *CRK10*, we used primers G700-SiRI and G700-SiBam to amplify a 500-bp fragment from the 5’-end of *CRK10*. The PCR product was processed the same way as the *CRK6* fragment for generating the end product Ubi-dsG700/C4300 construct. These constructs were also used to transform the NH1ox-11 line. Genotyping of CRK6Ri plants used primers G690-SiRI and Ubi-1 primers; genotyping of CRK10Ri plants used G700-SiRI and Ubi-1 primers.

A full-length, 2 kb *CRK10* cDNA was amplified with primers G700-3 and G700-8 from Nipponbare and cloned into pENTR/D. To generate an inducible *CRK10* construct in the GVG-DEX system to overexpress *CRK10*, the *CRK10* cDNA was subcloned into vector TA7002/GA by Gateway recombination, creating construct GVG-G700. Genotyping of the GVG-G700 construct in transgenic lines used primers Hyg-3 and Hyg-4, targeting the hygromycin selection marker.

For silencing of *NH1*, an RNAi construct was generated using the GVG-DEX inducible vector. A 500 bp *NH1* 5’-cDNA fragment was excised from *NH1* cDNA with EcoRI and SalI. This fragment was ligated with a Gus spacer digested with EcoRI into the pENTR/L16 vector. The resulting construct was recombined with a Gateway compatible pTA7002 binary vector to generate RNAi construct GVG-NH1Ri targeting *NH1*.

### Real time quantitative RT-PCR

Total RNA was extracted using the Trizol reagent (Invitrogen) and purified with spin-column (NucleoBond). One to five μg of total RNA each sample was used to synthesize cDNA for real time RT-PCR.

To assess the expression level of *CRK6*, primers G690-Q1a and G690-Q2 or primers G690-Q1b and G690-Q2 were used. These primers were determined to be specific to the *CRK6* gene. For *CRK10*, primers G700-RT3 and G700-RT5 were used for real time RT-PCR.

### BTH and DEX applications to plants

BTH was applied to rice leaves in a greenhouse in the form of a foliar spray at a concentration of 1 mM in the form of Actigard (Syngenta). DEX was dissolved in DMSO and diluted to 100 μM in 0.05% Tween 20 and applied by foliar spray.

### Electrophoresis mobility shift assay (EMSA)

For the EMSA assay, a probe was generated via annealing two oligonucleotides containing the putative TGA binding site. The top oligonucleotide contains biotin at the 5’end. Detection of biotin on the probe by streptavidin was carried out using a Chemiluminescent Nucleic Acid Detection Module (Thermo Scientific, Rockford, IL).

### Statistical analysis

Statistical analysis was carried out using the JMP Pro 10 statistics program.

### Protein expression in *E*. *coli* and purification

Protein expression in E. coli BL21 cells and purification of the fusion protein was carried out according to Chen et al. [56].

### Kinase activity assay

Kinase activity assay was conducted as described by Chen et al. [56].

## Supporting Information

S1 Table The nucleotide sequences of the primers used in this study are provided in the table. (DOC)

S1 Fig Genes in the 88-kb region deleted in the *snim1* mutant. A schematic diagram depicting the positions of the 11 annotated, expressed genes contained in the 88 kb region on chromosome 7 that is deleted in mutant *snim1*. The six *CRK*-encoding genes are highlighted as filled bars. *CRK6* is g35690 and *CRK10* is g35700.
(PPT)

S2 Fig Complementation with genes in the *snim1* deletion. Each gene, including the coding region, promoter (approximately 1.5 kb upstream of the start codon), and 3’ (500 bp) regions, was amplified, confirmed by sequencing, and cloned into binary vector C4300. T0 transgenic plants were generated by transforming mutant *snim1* with each individual gene in the 88-kb region deleted in *snim1*. T0 plants were inoculated with *Xoo* and lesion lengths measured and recorded 14 days after inoculation. Each bar represents the average and standard deviation of 2 to 8 leaves.
(PPT)

S3 Fig A phylogenetic tree of rice and Arabidopsis CRK proteins. Forty-five rice CRK protein sequences were retrieved from the MSU rice database RGAP V7. Forty four Arabidopsis CRK protein sequences are included in the tree construction. Multiple alignments with the ClustalX program were performed; bootstrapping was preformed 1000 times to generate the tree. The tree is viewed using FigTree v1.4.2.
(PPT)

S4 Fig *CRK6* and *CRK10* are silenced in the CRK6Ri and CRK10Ri lines. RNA was extracted from independent CRK6Ri and CRK10Ri lines as labeled under each bar. *CRK6* RNA levels were determined by running real time RT-PCR with primers G690-Q1a and G690-Q2, which are specific to the *CRK6* gene. Real time RT-PCRs were also carried out with primers G690-Q1b and G690-Q2 to confirm the above PCR results. *CRK10* RNA levels were assessed with primers G700-RT3 and G700-RT5, which are specific to the *CRK10* gene. RNA levels of g35580 (G580), g35650 (G650), g35660 (G660), and g35680 (G680) were also assessed in CRK6Ri #3 & #10 and CRK10Ri #4 & #13. Each bar represents the average and standard deviation of three replicates. The letters above each bar show the statistical groupings using the student T-test on each pair based on the 5% significance level.
(PPT)

S5 Fig The CRK10Ri construct cosegregates with enhanced susceptibility in progeny. Segregating progeny were genotyped for the presence of the CRK10Ri transgene. Those containing the transgene colored in green and the null segregants colored in orange. Progeny plants and the NH1ox parent were inoculated with *Xoo*. Lesion lengths were measured two weeks after inoculation. The inoculation results of three parental lines are presented. Each bar represents the average lesion length and standard deviation of all inoculated leaves from one plant. The letters above each bar show the statistical groupings using the student T-test on each pair based on the 5% significance level within the progeny of each line plus control.
(PPT)

S6 Fig The CRK6Ri construct cosegregates with enhanced susceptibility. Segregating progeny were genotyped for the presence of the CRK6Ri transgene. Those containing the transgene colored in green and the null segregants colored in orange. Progeny plants were inoculated with *Xoo* together with the NH1ox parent. Lesion lengths were measured two weeks after inoculation. The inoculation results of four lines are presented. Each bar represents the average lesion length and standard deviation of all inoculated leaves from one plant. The letters above each bar show the statistical groupings using the student T-test on each pair based on the 5% significance level within the progeny of each line plus control.
(PPT)

S7 Fig A 2-nucleotide deletion causes a frameshift in the *CRK10* gene of the *crk10*/Kitaake mutant. The sequences of the *crk10*/Kitaake mutant and the wild type Kitaake parent are aligned in the Integrative Genome Viewer program. A 2-nucleotide deletion is revealed in the reads of the *crk10*/Kitaake mutant.
(PPT)

S8 Fig The GVG-CRK10 construct cosegregates with enhanced resistance. Segregating progeny were genotyped for the presence of the GVG-CRK10 transgene. Those containing the transgene are presented in filled bars and the null segregants in open bars. Progeny plants were inoculated with *Xoo* together with the Kitaake control after DEX induction. Lesion lengths were measured two weeks after inoculation. The results of four lines from one inoculation and another line from another inoculation are presented. Each bar represents the average lesion length and standard deviation of all inoculated leaves from one plant. Progeny of lines #3, #4, #16, and #32 are compared together with the Kitaake control. Progeny of line #21 were compared with its own Kitaake control separately due to the different inoculation time. The letters above each bar show the statistical groupings using the student T-test on each pair based on the 5% significance level within the progeny of each line plus control.
(PPT)

S9 Fig *Xoo* inoculation results of transgenic rice plants overexpressing *CRK6*. *CRK6* overexpression transgenic lines carrying the *CRK6* gene driven by the maize *Ubi-1* promoter were generated in the Kitaake genetic background. (A) T0 plants were inoculated with *Xoo* and lesion lengths recorded 14 days after inoculation. Fourteen T0 lines are presented. Each bar represents the average and standard deviation of at least 5 leaves. (B) Four *CRK6* overexpression lines were tested for *CRK6* expression levels compared to the Kitaake control. Each bar represents three replicates.
(PPT)

S10 Fig BTH treatment, but not elevated *NH1* levels, induces *CRK6* expression. Kitaake (Kit) and nNH1 plants were treated with 1mM BTH and leaf samples taken at time points 1, 4, 8, 24, and 48 hours after treatment. The nNH1 samples were compared with the Kit control at each time point for analysis.
(PPT)

S11 Fig CRK6 kinase activity and CRK6 and CRK10 kinase domain sequence alignment. (A) The CRK6 kinase domain was fused to the His:Nus protein and expressed in *E coli* BL21 cells. The fusion protein was purified using Ni-NTA resins. A negative control containing a change from aspartate to asparagine at amino acid 488 was also expressed and purified. Kinase activity assay was performed in parallel for the two proteins. (B) The kinase domains of CRK6 and CRK10 are aligned using Geneious to display their similarity.
(PPT)

## References

[1] Ronald P. C. & Beutler B. (2010). Plant and Animal Sensors of Conserved Microbial Signatures. Science 330: 1061–1064 (2010). 10.1126/science.1189468 21097929

[2] Maekawa T., Kufer T.A. and Schulze-Lefert P. (2011). NLR functions in plant and animal immune systems: so far and yet so close. Nature Immunol.12: 817–826. 21852785 10.1038/ni.2083

[3] Vlot A.C., Dempsey D.A. and Klessig D.F. (2009). Salicylic acid, a multifaceted hormone to combat disease. Annu. Rev. Phytopathol. 47: 177–206. 10.1146/annurev.phyto.050908.135202 19400653

[4] Durrant W.E. and Dong X. Systemic acquired resistance. (2004). Annu. Rev. Phytopathol. 42: 185–209. 15283665 10.1146/annurev.phyto.42.040803.140421

[5] Ward E.R., Uknes S.J., Williams S.C., Dincher S.S., Wiederhold D.L., Alexader D.C., Ahl-Goy P., Metraux J.P., and Ryals J.A. (1991). Coordinate gene activity in response to agents that induce systemic acquired resistance. Plant Cell 3: 1085–1094. 12324583 10.1105/tpc.3.10.1085 PMC160074

[6] Friedrich L., Lawton K., Ruess W., Masner P, Speckner N., Gt Rella M., Meier B., Dinher S., Staub T., Uknes S., Metraux J.-P., Kessman H., and Ryals J. A. (1996). Benzothiadiazole derivative induces systemic acquired resistance in tobacco. Plant J. 9: 61–70.

[7] Yoshioka K., Nakashita H., Klessig D.F., and Yamaguchi I. (2001). Probenazole induces systemic acquired resistance in *Arabidopsis* with a novel type of action. Plant J. 25: 149–157. 11169191 10.1046/j.1365-313x.2001.00952.x

[8] Gorlach J., Volrath S., Knauf-Beiter G., Hengy G., Beckhove U., Kogel K.-H., Oostendorp M., Staub T., Ward E., Kessmann H., and Ryals J. (1996). Benzothiadiazole, a novel class of inducers of systemic acquired resistance, activates gene expression and disease resistance in wheat. Plant Cell 8: 629–643. 8624439 10.1105/tpc.8.4.629 PMC161125

[9] Rohilla R., Singh U.S., and Singh R.L. (2002). Mode of action of acibenzolar-S-methyl against sheath blight of rice, caused by Rhizoctonia solani kuhn. Pest Manag. Sci., 58: 63–69. 11838287 10.1002/ps.423

[10] Shimono M., Sugano S., Nakayama A., Jiang C.J., Ono K., Toki S., and Takatsuji H. (2007). Rice WRKY45 plays a crucial role in benzothiadiazole-inducible blast resistance. Plant Cell 19: 2064–2076. 17601827 10.1105/tpc.106.046250 PMC1955718

[11] Cao H., Bowling S.A., Gordon A.S., and Dong X. (1994). Characterization of an Arabidopsis mutant that is nonresponsive to inducers of systemic acquired resistance. Plant Cell 6: 1583–1592. 12244227 10.1105/tpc.6.11.1583 PMC160545

[12] Delaney T.P., Friedrich L., and Ryals J.A. (1995). Arabidopsis signal transduction mutant defective in chemically and biologically induced disease resistance. Proc. Natl. Acad. Sci. 92: 6602–6606. 11607555 10.1073/pnas.92.14.6602 PMC41566

[13] Glazebrook J., Rogers E.E., and Ausubel F.M. (1996). Isolation of *Arabidopsis* mutants with enhanced disease susceptibility by direct screening. Genetics 143: 973–982. 8725243 10.1093/genetics/143.2.973 PMC1207353

[14] Ryals J., Weymann K., Lawton K., Friedrich L., Ellis D., Steiner H.-Y., Johnson J., Delaney T.P., Jesse T., Vos P., and Uknes S. (1997). The Arabidopsis NIM1 protein shows homology to the mammalian transcription factor inhibitor IκB. Plant Cell 9: 425–439. 9090885 10.1105/tpc.9.3.425 PMC156928

[15] Shah J., Tsui. F., and Klessig D.F. (1997). Characterization of a salicylic acid-insensitive mutant (*sai1*) of *Arabidopsis thaliana*, identified in a selective screen utilizing the SA-inducible expression of the tms2 gene. Mol. Plant Microbe Interact. 10: 69–78. 9002272 10.1094/MPMI.1997.10.1.69

[16] Cao H., Glazebrook J., Clarke J., Volko S., and Dong X. (1997). The Arabidopsis npr1 gene that controls systemic acquired resistance encodes a novel protein containing ankyrin repeats. Cell 88: 57–63. 9019406 10.1016/s0092-8674(00)81858-9

[17] Cao H., Li X. and Dong X. (1998). Generation of broad-spectrum disease resistance by overexpression of an essential regulatory gene in systemic acquired resistance. Proc. Natl. Acad. Sci. 95: 6531–6536. 9601001 10.1073/pnas.95.11.6531 PMC34547

[18] Friedrich L., Lawton K., Dietrich R., Willits M., Cade R., and Ryals J. (2001). NIM1 overexpression in Arabidopsis potentiates plant disease resistance and results in enhanced effectiveness of fungicides. Mol Plant Microbe Interact. 14: 1114–1124. 11551076 10.1094/MPMI.2001.14.9.1114

[19] Mou Z., Fan W., and Dong X. (2003). Inducers of plant systemic acquired resistance regulate NPR1 function through redox changes. Cell 113: 1–10. 12837250 10.1016/s0092-8674(03)00429-x

[20] Zhang Y., Fan. W., Kinkema M., Li X., and Dong X. (1999). Interaction of NPR1 with basic leucine zipper protein transcription factors that bind sequences required for salicylic acid induction of the PR-1 gene. Proc. Natl. Acad. Sci. USA 96: 6523–6528. 10339621 10.1073/pnas.96.11.6523 PMC26915

[21] Despres C., DeLong C., Glaze. S., Liu E., and Fobert P.R. (2000). The Arabidopsis NPR1/NIM1 protein enhances the DNA binding activity of a subgroup of the TGA family of bZIP transcription factors. Plant Cell 12: 279–290. 10662863 PMC139764

[22] Chern M., Fitzgerald H.A., Yadav R.C., Canlas P.E., Dong X., and Ronald P.C. (2001). Evidence for a disease-resistance pathway in rice similar to the NPR1-mediated signaling pathway in Arabidopsis. Plant J. 27: 101–113. 11489188 10.1046/j.1365-313x.2001.01070.x

[23] Fan W. and Dong X. (2002). *In vivo* interaction between NPR1 and transcription factor TGA2 leads to salicylic acid-mediated gene activation in Arabidopsis. Plant Cell 14: 1377–1389. 12084833 10.1105/tpc.001628 PMC150786

[24] Zhang Y., Tessaro M.J., Lassner M., and Li X. (2003). Knockout analysis of Arabidopsis transcription factors TGA2, TGA5, and TGA6 reveals their redundant and essential roles in systemic acquired resistance. Plant Cell 15: 2647–2653. 14576289 10.1105/tpc.014894 PMC280568

[25] Rochon A., Boyle P., Wignes T., Fobert P.R., and Després C. (2006) The coactivator function of Arabidopsis NPR1 requires the core of its BTB/POZ domain and the oxidation of C-terminal cysteines. Plant Cell 18: 3670–3685. 17172357 10.1105/tpc.106.046953 PMC1785396

[26] Boyle P., Su E.L., Rochon A., Shearer H.L., Murmu. J. Chu J.Y., Fobert P.R., and Despres C. (2009) The BTB/POZ domain of the Arabidopsis disease resistance protein NPR1 interacts with the repression domain of TGA2 to negate its function. Plant Cell 21: 3700–3713. 10.1105/tpc.109.069971 19915088 PMC2798319

[27] Wu Y., Zhang D., Chu J.Y., Boyle P., Wang Y., Brindle I.D., Luca V.D., and Despres C. (2012). The Arabidopsis NPR1 protein is a receptor for the plant defense hormone salicylic acid. Cell Rep. 1: 639–647. 10.1016/j.celrep.2012.05.008 22813739

[28] Fu Z.Q., Yan S., Saleh A., Wang W., Ruble J., Oka N., Mohan R., Spoel S.H., Tada Y., Zheng N., and Dong X. (2012). NPR3 and NPR4 are receptors for the immune signal salicylic acid in plants. Nature 486: 228–232. 10.1038/nature11162 22699612 PMC3376392

[29] Chern M., Fitzgerald H.A., Canlas P.E., Navarre D.A., and Ronald P.C. (2005). Over-expression of a rice NPR1 homolog leads to constitutive activation of defense response and hypersensitivity to light. Mol. Plant Microbe Interact. 18: 511–520. 15986920 10.1094/MPMI-18-0511

[30] Yuan Y., Zhong S., Li Q., Zhu Z., Lou Y., Wang L., Wang J., Wang M., Li Q., Yang D., and He Z. (2007). Functional analysis of rice NPR1-like genes reveals that OsNPR1/NH1 is the rice orthologue conferring disease resistance with enhanced herbivore susceptibility. Plant Biotechnol. J. 5: 313–324. 17309686 10.1111/j.1467-7652.2007.00243.x

[31] Weigel R.R., Pfitzner U.M. and Gatz C. (2005) Interaction of NIMIN1 with NPR1 modulates PR gene expression in Arabidopsis. Plant Cell, 17, 1279–1291. 15749762 10.1105/tpc.104.027441 PMC1088002

[32] Chern M., Canlas P.E., Fitzgerald H.A., Ronald P.C. (2005). Rice NRR, a negative regulator of disease resistance, interacts with Arabidopsis NPR1 and rice NH1. Plant J. 43, 623–635. 16115061 10.1111/j.1365-313X.2005.02485.x

[33] Hermann M., Maier F., Nasroor A., Hirth S., Pfitzner A.J.P., and Pfitzner U.M. (2013). The Arabidopsis NIMIN proteins affect NPR1 differentially. Front Plant Sci. 4:88 10.3389/fpls.2013.00088 23630533 PMC3624081

[34] Bai W., Chern M., Ruan D., Canlas P.E., Sze-To W.H., and Ronald P.C. (2011). Enhanced disease resistance and hypersensitivity to BTH by introduction of an NH1/OsNPR1 paralog. Plant Biotechnol. J. 9: 205–215. 10.1111/j.1467-7652.2010.00544.x 20561248

[35] Lorrain S., Valileau F., Balague C., and Roby D. (2003). Lesion mimic mutants: keys for deciphering cell death and defense pathways in plants. Trends Plant Sci. 8: 263–271. 12818660 10.1016/S1360-1385(03)00108-0

[36] Yin Z., Chen J., Zeng L., Goh M., Leung H., Khush G., and Wang G.-L. (2000). Characterizing rice lesion mimic mutants and identifying a mutant with broad-spectrum resistance to rice blast and bacterial blight. Mol. Plant-Microbe Interact. 13: 869–876. 10939258 10.1094/MPMI.2000.13.8.869

[37] Chern M., Bai W., Sze-To W.H., Canlas P.E., Bartley L.E., and Ronald P.C. (2012). A rice transient assay system identifies a novel domain in NRR required for interaction with NH1/OsNPR1 and inhibition of NH1-mediated transcriptional activation. Plant Methods 8: 6 10.1186/1746-4811-8-6 22353606 PMC3297495

[38] Bart R., Chern M., Vega-Sanchez M., Canlas P., and Ronald P.C. (2010). Rice Snl6, a cinnamoyl-CoA reductase-like gene family member, is required for NH1-mediated immunity to Xanthomonas oryzae pv. oryzae. PLoS Genet. 6: e1001123 10.1371/journal.pgen.1001123 20862311 PMC2940737

[39] Li G., Chern M., Jain R., Martin J.A., Schackwitz W.S., Jiang L., Vega-Sánchez M.E., Lipzen A.M., Barry K.W., Schmutz J., and Ronald P.C. (2016). Genome-wide sequencing of 41 rice mutated lines reveals diverse mutations induced by fast-neutron irradiation. Mol. Plant, In press. 10.1016/j.molp.2016.03.009 27018389

[40] Aoyama T. and Chua N.H. (1997). A glucocorticoid-mediated transcriptional induction system in transgenic plants. Plant J. 11: 605–612. 9107046 10.1046/j.1365-313x.1997.11030605.x

[41] Chern M., Bai W., Chen X., Canlas P.E., and Ronald P.C. (2013). Reduced expression of glycolate oxidase leads to enhanced disease resistance in rice. PeerJ. 1:e28 10.7717/peerj.28 23638363 PMC3628735

[42] Fitzgerald H.A., Canlas P.E., Chern M., and Ronald P.C. (2005). Alteration of TGA factor activity in rice results in enhanced tolerance to *Xanthomonas oryzae* pv. *oryzae*. Plant J. 43, 335–347. 16045470 10.1111/j.1365-313X.2005.02457.x

[43] Chern M., Bai W., Ruan D.. Oh T., Chen X., Ronald P.C. (2014) Interaction specificity and coexpression of rice NPR1 homologs 1 and 3 (NH1 and NH3), TGA transcription factors and Negative Regulator of Resistance (NRR) proteins. BMC Genomics 15: 461 10.1186/1471-2164-15-461 24919709 PMC4094623

[44] Chen Z. (2001) A superfamily of proteins with novel cysteine-rich repeats. Plant Physiol. 126: 473–476. 11402176 10.1104/pp.126.2.473 PMC1540112

[45] Burdiak P, Rusaczonek A, Witon D, Glow D, Karpinski S. (2015) Cysteine-rich receptor kinase CRK5 as a regulator of growth, development, and ultraviolet radiation responses in Arabidopsis thaliana. J. Exp. Bot. 66:3325–3337. 10.1093/jxb/erv143 25969551 PMC4449547

[46] Lozano R., Hamblin M.T., Prochnik S., and Jannink J.-L. (2015). Identification and distribution of the NBS-LRR gene fanily in the Cassava genome. BMC Genomics 16:360 10.1186/s12864-015-1554-9 25948536 PMC4422547

[47] Wang G.L., Ruan D.L., Song W.Y., Sideris S., Chen L., Pi L.Y., Zhang S., Zhang Z., Fauquet C., Gaut B.B., Whalen M.C., and Ronald P.C. (1998). Xa21D encodes a receptor-like molecule with a leucine-rich repeat domain that determines race-specific recognition and is subject to adaptive evolution. Plant Cell 10: 765–779. 9596635 10.1105/tpc.10.5.765 PMC144027

[48] Chen K., Du L., and Chen Z. (2003). Sensitization of defense responses and activation of programmed cell death by a pathogen-induced receptor-like protein kinase in Arabidopsis. Plant Mol. Biol. 53: 61–74. 14756307 10.1023/B:PLAN.0000009265.72567.58

[49] Acharya B.R., Raina S., Maqbool S.B., Jagadeeswaran G., Mosher S.L., Appel H.M., Schultz J.C., Klessig D.F. and Raina R. (2007). Overexpression of CRK13, an Arabidopsis cysteine-rich receptor-like kinase, results in enhanced resistance to Pseudomonas syringae. Plant J. 50: 488–499. 17419849 10.1111/j.1365-313X.2007.03064.x

[50] Zhang X., Han X., Shi R., Yang G., Qi L., Wang R., and Li G.(2013) Arabidopsis cycteine-rich receptor-like kinase 45 positively regulates disease resistance to Pseudomonas syringae. Plant Physiol. Biochem. 73: 383–391. 10.1016/j.plaphy.2013.10.024 24215930

[51] Wrzaczek M., Brosche M., Salojarvi J., Kangasjarvi S., Idanheimo N., Mersmann S., Robatzek S., Karpinski S., Karpinska B., and Kangasjarvi J. (2010). Transcriptional regulation of the CRK/DUF26 group of receptor-like protein kinases by ozone and plant hormones in Arabidopsis. BMC Plant Biol. 10: 95 10.1186/1471-2229-10-95 20500828 PMC3095361

[52] Ederli L., Madeo L., Calderini O., Gehring C., Moretti C., Buonaurio R., Paolocci F. and Pasqualini S. (2011). The Arabidopsis thaliana cysteine-rich receptor-like kinase CRK20 modulates host responses to Pseudomonas syringae pv. tomato DC3000 infection. J. Plant Physiol. 168: 1784–1794. 10.1016/j.jplph.2011.05.018 21742407

[53] Yeh Y.-H., Chang Y.-H., Huang P.-Y., Huang J.-B. and Zimmerli L. (2015). Enhanced Arabidopsis pattern-triggered immunity by overexpression of cysteine-rich receptor-like kinases. Front. Plant Sci. 6:322 10.3389/fpls.2015.00322 26029224 PMC4429228

[54] Kauffman H.E., Reddy A.P.K., Hsieh S.P.V., and Marca S.D. (1973). An improved technique for evaluation of resistance of rice varieties to Xanthomonas oryzae. Plant Disease Reporter 57: 537–541.

[55] Pruitt R.N., Schwessinger B., Joe A., Thomas N., Liu F., Albert M., Robinson M., Chan L., Luu D., Chen H., Bahar O., Daudi A., De Vleesschauwer D., Caddell D., Zhang W., Zhao X., Li X., Heazelwood J., Ruan D., Majumder D., Chern M., Kalbacher H., Sonti R., Petzold C., Liu C., Brodbelt J., Felix G., Ronald P.C. (2015). The rice immune receptor XA21 recognizes a tyrosine sulfated peptide from a Gram-negative bacterium. Science Advances Vol. 1, no. 6, e1500245 10.1126/sciadv.1500245 PMC4646787 26601222

[56] Chen X., Zuo S., Schwessinger B., Chern M., Canlas P.E., Ruan D., Zhou X., Wang J., Daudi A., Petzold C.J., Heazlewood J.L., Ronald P.C. (2014). An XA21-associated kinase (OsSERK2) regulates immunity mediated by the XA21 and XA3 immune receptors. Mol. Plant 7:874–892. 10.1093/mp/ssu003 24482436 PMC4064043

