# Correction: A Genetic Screen Identifies a Requirement for Cysteine-Rich-Receptor-Like Kinases in Rice NH1 (OsNPR1)-Mediated Immunity

**DOI:** 10.1371/journal.pgen.1006182

**Published:** 2016-07-06

**Authors:** Mawsheng Chern, Qiufang Xu, Rebecca S. Bart, Wei Bai, Deling Ruan, Wing Hoi Sze-To, Patrick E. Canlas, Rashmi Jain, Xuewei Chen, Pamela C. Ronald

There is information missing from the Funding section, specifically regarding the United States Department of Energy and the Lawrence Berkeley National Laboratory. The correct statement is as follows: This work was supported by a grant to PCR from NIH (GM59962) and NSF (1237975). This work was part of the DOE Joint BioEnergy Institute (http://www.jbei.org/) supported by the U. S. Department of Energy, Office of Science, Office of Biological and Environmental Research, through contract DE-AC02-05CH11231 between Lawrence Berkeley National Laboratory and the U. S. Department of Energy. The United States Government retains and the publisher, by accepting the article for publication, acknowledges that the United States Government retains a non-exclusive, paid-up, irrevocable, world-wide license to publish or reproduce the published form of this manuscript, or allow others to do so, for United States Government purposes. The funders had no role in study design, data collection and analysis, decision to publish, or preparation of the manuscript.
