# Supplementary material for: A Genetic Screen Identifies a Requirement for Cysteine-Rich–Receptor-Like Kinases in Rice NH1 (OsNPR1)-Mediated Immunity
Source: PLoS Genet. 2016 May 13;12(5):e1006049. doi: 10.1371/journal.pgen.1006049 (PMC4866720; doi:10.1371/journal.pgen.1006049)
Supplement: S1 Table — (DOC) [file pgen.1006049.s001.doc]

**Supplementary Table 1. Primer sequences**

| Primer name | Sequence |
| --- | --- |
| G580-1 | CACCTATTGTTTGAGCTACATGTGGACATCA |
| G580-3 | ACCAGTGAGTACACTACTCTATTC |
| G600-1 | CACCTGCGACTTCTCCGTTAGCTGTCGG |
| G600-2 | CTTCACCATCCGCCACTAAAAAGC |
| G610-1 | CACCGTGACGTGTCTGTCTCACTG |
| G610-3 | GGAATAATAACAAACAGGCCTAACACCC |
| G620-1a | CACCTCATGGAGGAGTCGCGGA |
| G620-2a | TCAGGACGCTGGGGTGAAAG |
| G630-1 | CACCTAAGCATGTCTAAGCAATTCCTAGTCCA |
| G630-2 | GATCTTCAGCCGTGATTCTTCATGG |
| G640-1 | CACCGTATTTCACCTGTAAACTGCGAGATG |
| G640-2 | ACACAAGATTGGCTACATGGGCATCGAGA |
| G650-1 | CACCGAATTTTGCTCCTTTCTATATCAGCTTCAATGG |
| G650-2 | CCTTTATTGTGCGCACAAATACAGGT |
| G660-1 | CACCTAGGCAATAGAGAATCGGATAGTGA |
| G660-2 | TTCGGGCAGTGTAGAGTAGATGTTG |
| G680-1 | CACCCGGTCCAGAAATCCGGATTTCCT |
| G680-3 | GACCGATACCAGTACCACTCGG |
| G690-1 | CACCTCCCTATTCTCAGTTCTAGAACCAAGCA |
| G690-3 | GTGCGTTAAAAAGTTCAAAGTCGTATCTCCGGT |
| G700-1 | CACCTCCCTTCCATGCTTCTCAAACC |
| G700-2 | GGTCAGGATCTTGCTTGTAGGGA |
| G690-SiRI | TTTGAATTCACCAGGTCAACCTCGACCTC |
| G690-SiBam | TTGGATCCAGTTGCCCGATCACCGTCGAGA |
| G700-SiRI | TTTGAATTCACTACACGGAGCACGGCACG |
| G700-SiBam | TTTGGATCCATGTCTGGCGTGCACTGC |
| G700-3 | CACCATGTCCATGGCCTGCTACTACC |
| G700-8 | TCTTGAGTTGTGTGGGTTC |
| Hyg-3 | TCCACTATCGGCGAGTACTTCTACACA |
| Hyg-4 | CACTGGCAAACTGTGATGGACGAC |
| G690-3b | GGATTCATCTCGGCACAAGCTCTGTG |
| G690-4c | CACCATTACCATCGCCAGCACA |
| NH2-3 | AAGAATTCCTCGATTCCGACCTCGACT |
| NH2-4 | AAGGATCCGTCGCTGGAACAGCGAGA |
| NH3-1 | AAGAATTCAGATCTGAGACGTCCACCATAAG |
| NH3-2 | GTGGCTGCAGCCGTCGTCCA |
| G690-Q1a | CCAAAGAATTCAGCGGGAGG |
| G690-Q2 | GTCGCCGATGGCGAAGGC |
| G690-Q1b | TCGACGGTGATCGGGCAACT |
| G700-RT3 | TTTGGCTCCTACGGTTCTGAC |
| G700-RT5 | CACAGAGTAGCCCAATGTGGA |
| G690-RT1 | GATAGTGGGCAAGATGTTGATCTC |
| G690-RT2 | TGGATAGCGTTCTGAATACGGA |
| G700-RT4 | TGCACCTTAGCTAGCAGTAGCA |
| G610-2 | GCTACTAAGTTGGTGGATTACCTAGC |
| G610-10 | CACCATGATGGTGAAGAAAAAAACCTCTTGGAGC |
| Ubi-1 | TGATATACTTGGATGATGGCA |
| G700-P2 | CACCTTAGTGAACTATCACTTACCGTAG |
| G700-P3 | CATGTGTGCCATGTCTGAGAC |
| G580-qRT1 | TCCTCCTCCTCCGTGTCCATG |
| G580-qRT2 | CTTGAGGTTGGCTTGGTAGGTG |
| G650-qRT1 | TCCTCCTCCACGTCCACCTC |
| G650-qRT2 | TGCTGTTCTCCGAGTAGCGG |
| G660-qRT1 | ACGCCGACGAAATCTCACTCC |
| G660-qRT2 | CGGCGGTGTCGCACATCTGC |
| G680-qRT1 | TCCTCGAGCAGCTAGACCAC |
| G680-qRT2 | GAGGCGCGTGGAGGAACACC |
