# Supplementary figures and images for: A Genetic Screen Identifies a Requirement for Cysteine-Rich–Receptor-Like Kinases in Rice NH1 (OsNPR1)-Mediated Immunity
Source: PLoS Genet. 2016 May 13;12(5):e1006049. doi: 10.1371/journal.pgen.1006049 (PMC4866720; doi:10.1371/journal.pgen.1006049)

## Slide 1
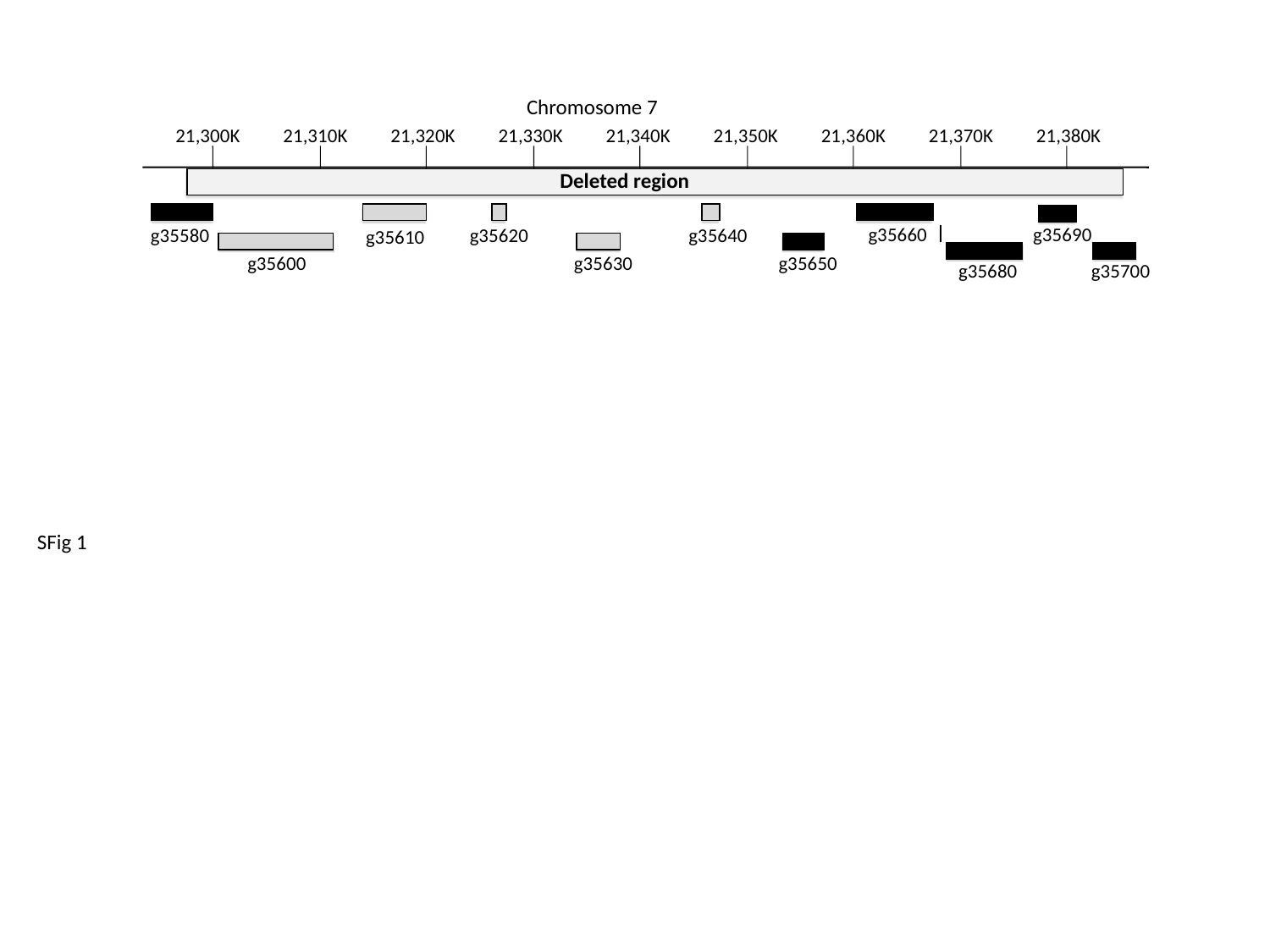

Chromosome 7
21,300K
21,310K
21,320K
21,330K
21,340K
21,350K
21,360K
21,370K
21,380K
Deleted region
g35660
g35690
g35580
g35620
g35640
g35610
g35600
g35630
g35650
g35680
g35700
SFig 1

Supplement: S1 Fig — A schematic diagram depicting the positions of the 11 annotated, expressed genes contained in the 88 kb region on chromosome 7 that is deleted in mutant snim1. The six CRK-encoding genes are highlighted as filled bars. CRK6 is g35690 and CRK10 is g35700. (PPT) [file pgen.1006049.s002.ppt]

## Slide 1
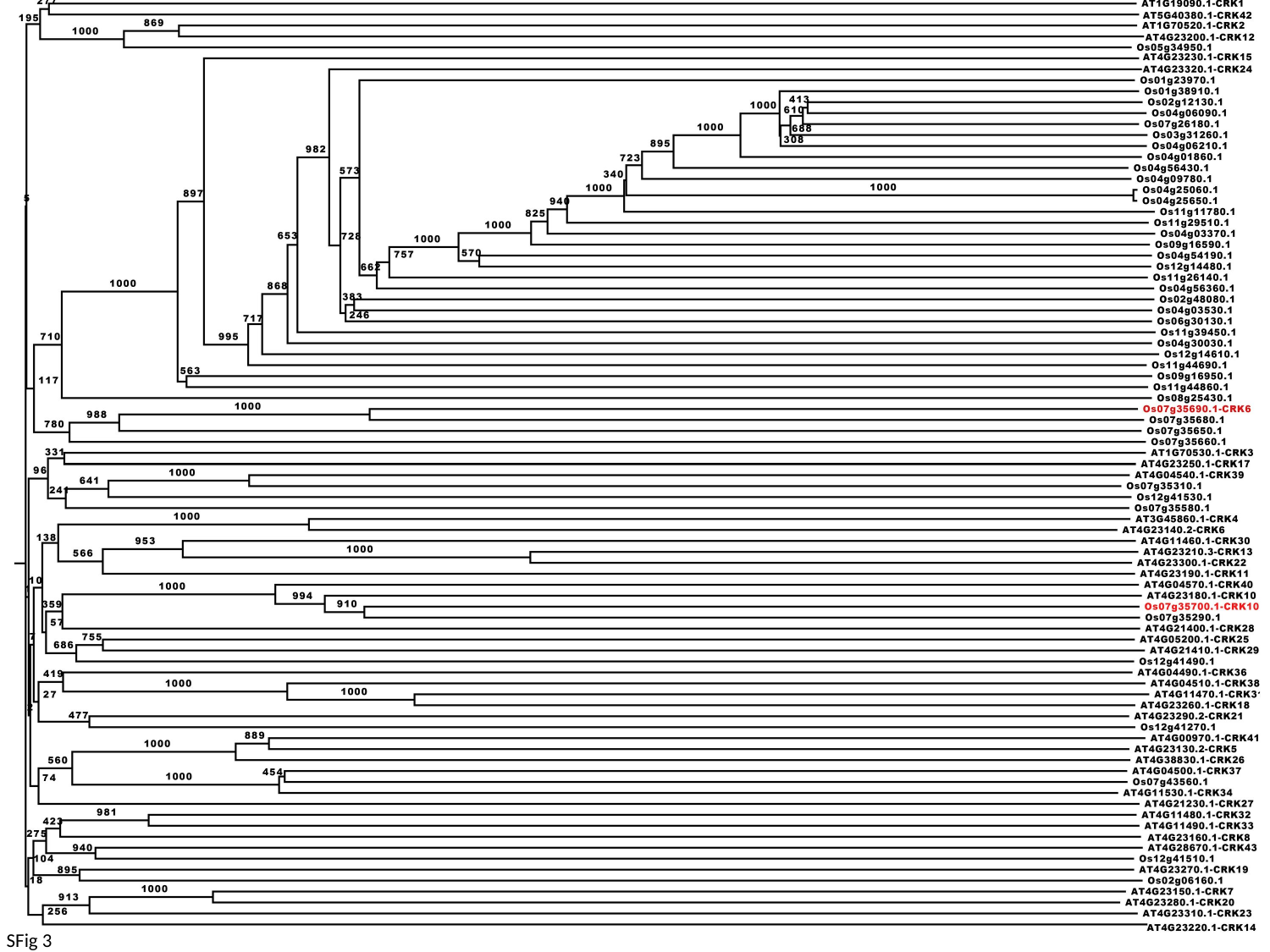

SFig 3

Supplement: S3 Fig — Forty-five rice CRK protein sequences were retrieved from the MSU rice database RGAP V7. Forty four Arabidopsis CRK protein sequences are included in the tree construction. Multiple alignments with the ClustalX program were performed; bootstrapping was preformed 1000 times to generate the tree. The tree is viewed using FigTree v1.4.2. (PPT) [file pgen.1006049.s004.ppt]

## Slide 1
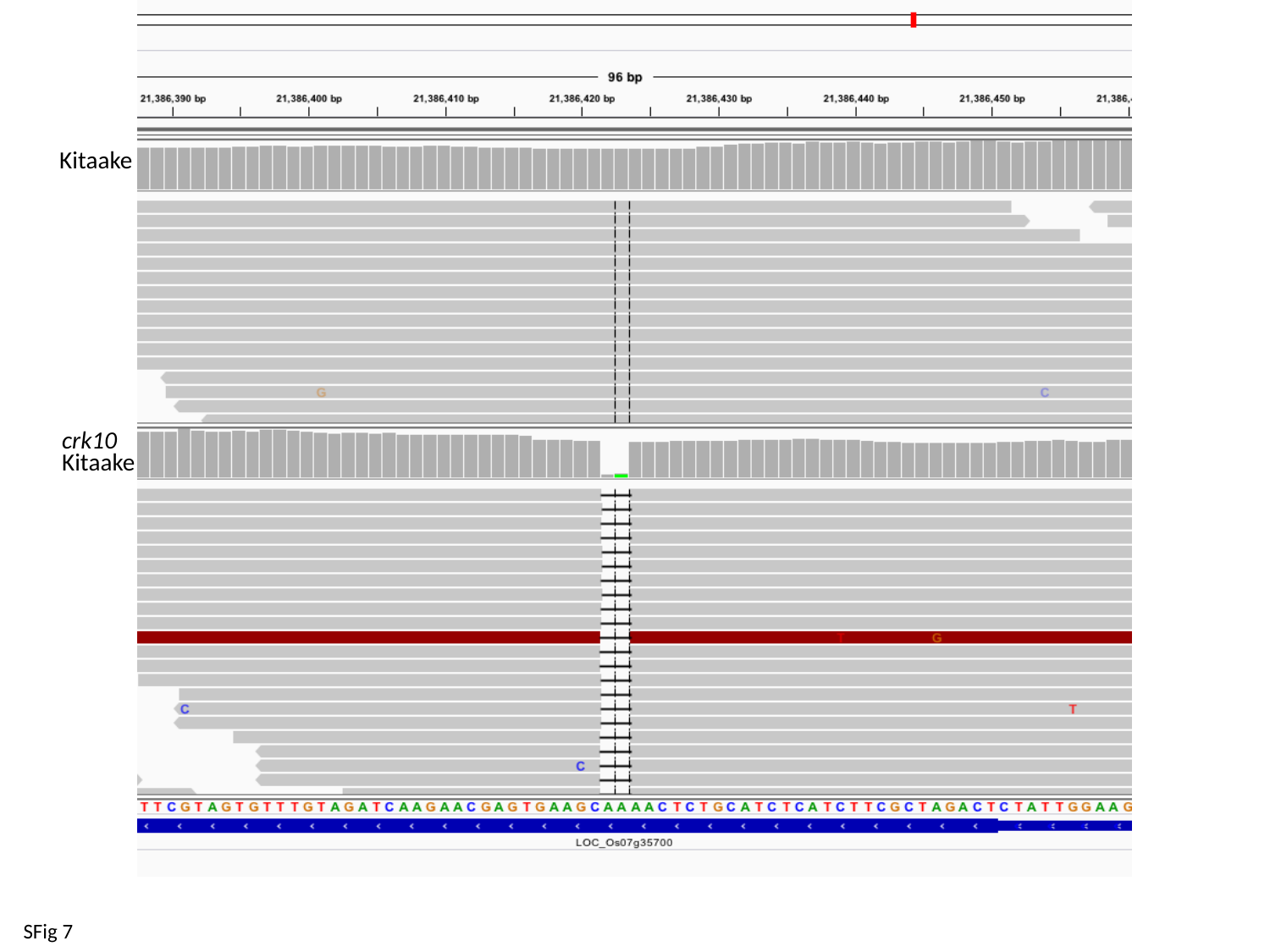

Kitaake
crk10
Kitaake
SFig 7

Supplement: S7 Fig — The sequences of the crk10/Kitaake mutant and the wild type Kitaake parent are aligned in the Integrative Genome Viewer program. A 2-nucleotide deletion is revealed in the reads of the crk10/Kitaake mutant. (PPT) [file pgen.1006049.s008.ppt]

## Slide 1
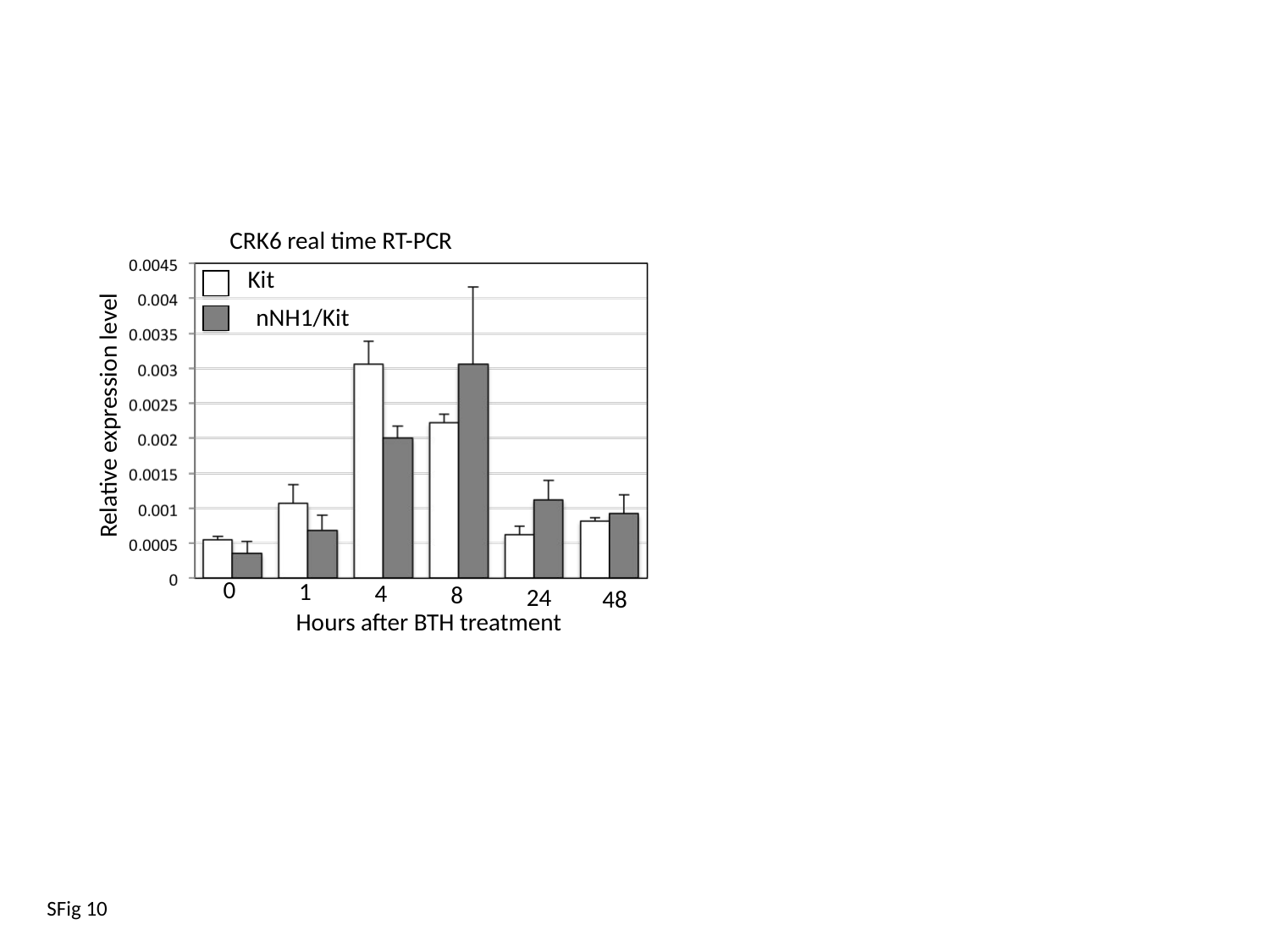

CRK6 real time RT-PCR
Kit
nNH1/Kit
Relative expression level
0
1
4
8
24
48
Hours after BTH treatment
SFig 10

Supplement: S10 Fig — Kitaake (Kit) and nNH1 plants were treated with 1mM BTH and leaf samples taken at time points 1, 4, 8, 24, and 48 hours after treatment. The nNH1 samples were compared with the Kit control at each time point for analysis. (PPT) [file pgen.1006049.s011.ppt]
