# Supplementary material for: A Genetic Screen Identifies a Requirement for Cysteine-Rich–Receptor-Like Kinases in Rice NH1 (OsNPR1)-Mediated Immunity
Source: PLoS Genet. 2016 May 13;12(5):e1006049. doi: 10.1371/journal.pgen.1006049 (PMC4866720; doi:10.1371/journal.pgen.1006049)
Supplement: S2 Fig — Each gene, including the coding region, promoter (approximately 1.5 kb upstream of the start codon), and 3’ (500 bp) regions, was amplified, confirmed by sequencing, and cloned into binary vector C4300. T0 transgenic plants were generated by transforming mutant snim1 with each individual gene in the 88-kb region deleted in snim1. T0 plants were inoculated with Xoo and lesion lengths measured and recorded 14 days after inoculation. Each bar represents the average and standard deviation of 2 to 8 leaves. (PPT) [file pgen.1006049.s003.ppt]

## Slide 1
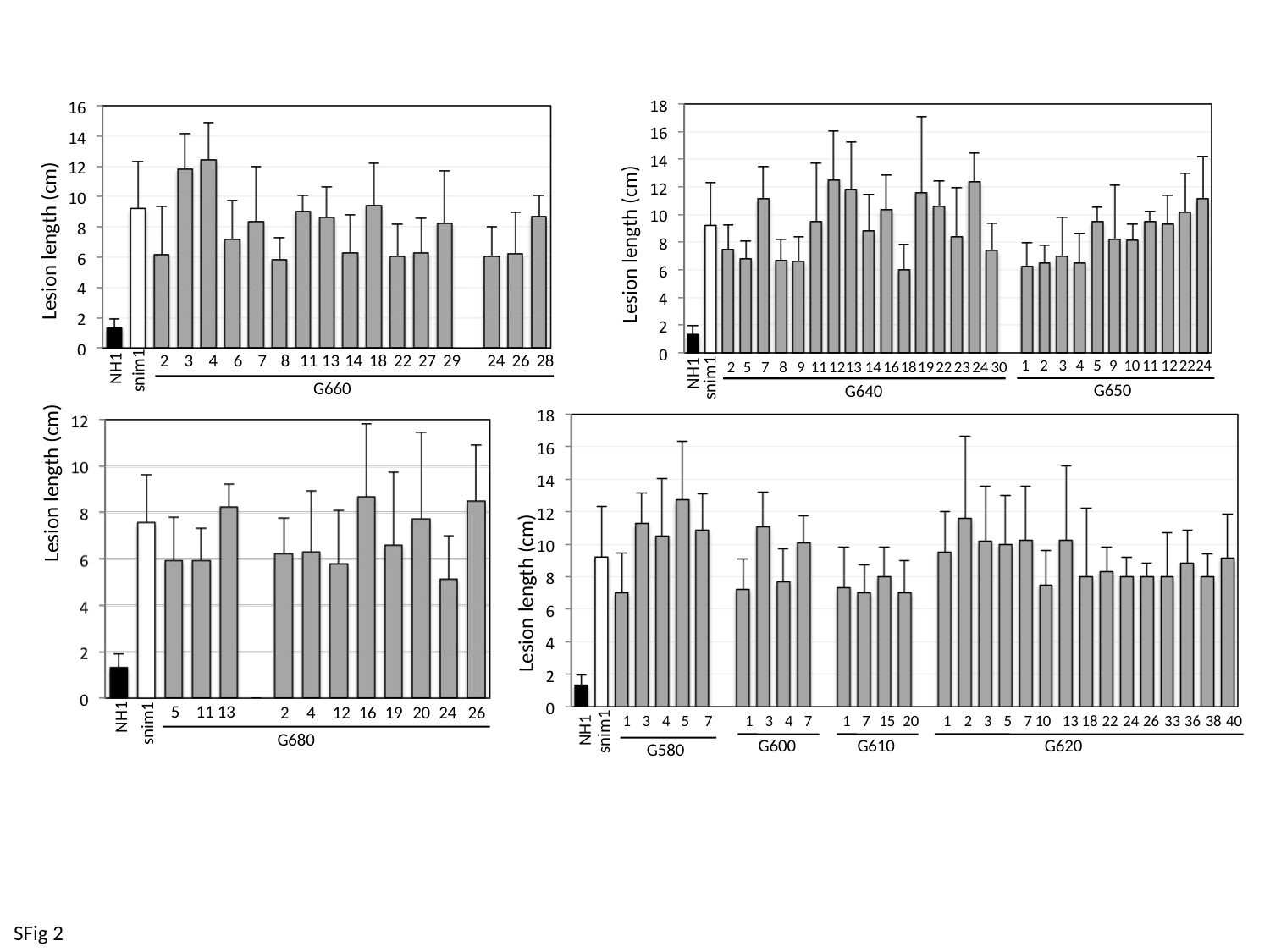

Lesion length (cm)
1
2
3
4
5
9
10
11
12
22
24
2
5
7
8
9
11
12
13
14
16
18
19
22
23
24
30
NH1
snim1
G650
G640
Lesion length (cm)
2
3
4
6
7
8
11
13
14
18
22
27
29
24
26
28
NH1
snim1
G660
Lesion length (cm)
5
11
13
2
4
12
16
19
20
24
26
NH1
snim1
G680
Lesion length (cm)
1
3
4
5
7
1
3
4
7
1
7
15
20
1
2
3
5
7
10
13
18
22
24
26
33
36
38
40
NH1
snim1
G600
G610
G620
G580
SFig 2
