# Supplementary material for: A Genetic Screen Identifies a Requirement for Cysteine-Rich–Receptor-Like Kinases in Rice NH1 (OsNPR1)-Mediated Immunity
Source: PLoS Genet. 2016 May 13;12(5):e1006049. doi: 10.1371/journal.pgen.1006049 (PMC4866720; doi:10.1371/journal.pgen.1006049)
Supplement: S4 Fig — RNA was extracted from independent CRK6Ri and CRK10Ri lines as labeled under each bar. CRK6 RNA levels were determined by running real time RT-PCR with primers G690-Q1a and G690-Q2, which are specific to the CRK6 gene. Real time RT-PCRs were also carried out with primers G690-Q1b and G690-Q2 to confirm the above PCR results. CRK10 RNA levels were assessed with primers G700-RT3 and G700-RT5, which are specific to the CRK10 gene. RNA levels of g35580 (G580), g35650 (G650), g35660 (G660), and g35680 (G680) were also assessed in CRK6Ri #3 & #10 and CRK10Ri #4 & #13. Each bar represents the average and standard deviation of three replicates. The letters above each bar show the statistical groupings using the student T-test on each pair based on the 5% significance level. (PPT) [file pgen.1006049.s005.ppt]

## Slide 1
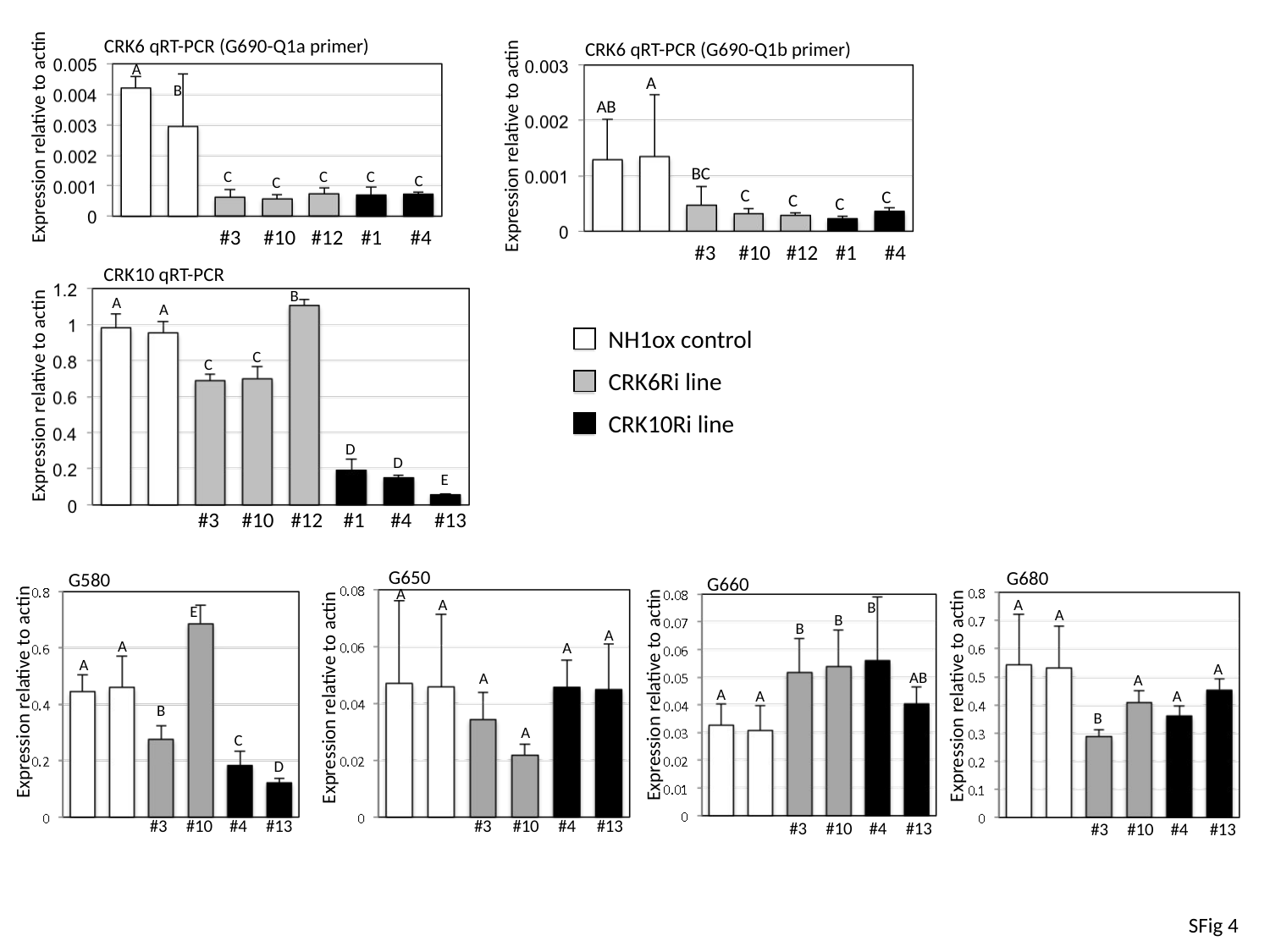

CRK6 qRT-PCR (G690-Q1a primer)
A
B
C
C
C
C
C
#3
#10
#12
#1
#4
Expression relative to actin
CRK6 qRT-PCR (G690-Q1b primer)
A
AB
BC
C
C
C
C
#3
#10
#12
#1
#4
Expression relative to actin
CRK10 qRT-PCR
B
A
A
NH1ox control
C
C
CRK6Ri line
Expression relative to actin
CRK10Ri line
D
D
E
#3
#10
#12
#1
#4
#13
G650
A
A
A
A
A
Expression relative to actin
A
#13
#3
#10
#4
G680
G580
E
A
A
Expression relative to actin
B
C
D
#3
#10
#4
#13
G660
A
B
A
B
B
A
AB
A
Expression relative to actin
A
Expression relative to actin
A
A
B
#3
#10
#4
#13
#3
#10
#4
#13
SFig 4
