# Supplementary material for: A Genetic Screen Identifies a Requirement for Cysteine-Rich–Receptor-Like Kinases in Rice NH1 (OsNPR1)-Mediated Immunity
Source: PLoS Genet. 2016 May 13;12(5):e1006049. doi: 10.1371/journal.pgen.1006049 (PMC4866720; doi:10.1371/journal.pgen.1006049)
Supplement: S5 Fig — Segregating progeny were genotyped for the presence of the CRK10Ri transgene. Those containing the transgene colored in green and the null segregants colored in orange. Progeny plants and the NH1ox parent were inoculated with Xoo. Lesion lengths were measured two weeks after inoculation. The inoculation results of three parental lines are presented. Each bar represents the average lesion length and standard deviation of all inoculated leaves from one plant. The letters above each bar show the statistical groupings using the student T-test on each pair based on the 5% significance level within the progeny of each line plus control. (PPT) [file pgen.1006049.s006.ppt]

## Slide 1
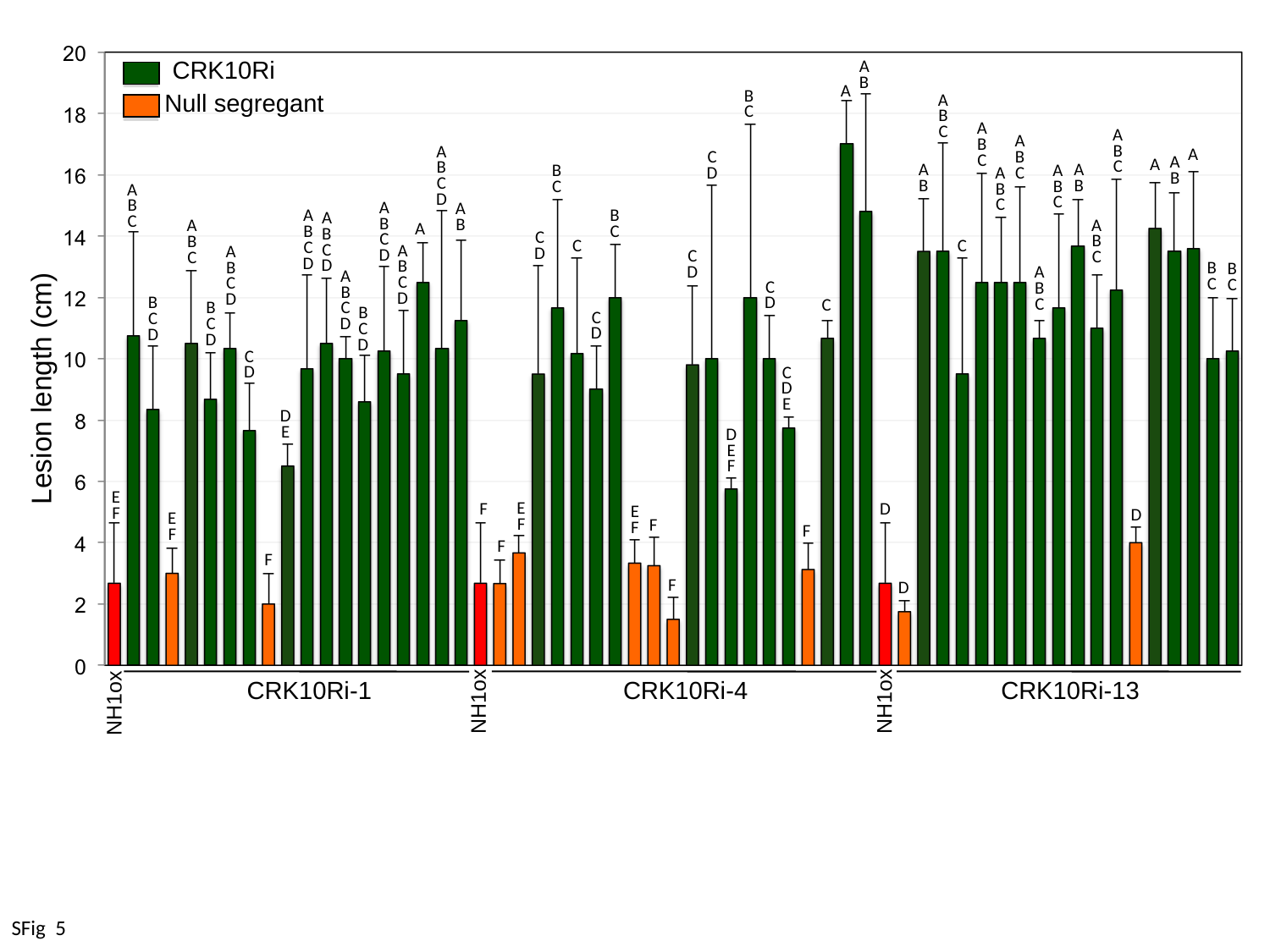

CRK10Ri
Null segregant
A
B
A
B
C
A
B
C
A
B
C
A
B
C
A
B
C
A
B
C
D
A
C
D
A
B
A
A
B
A
B
A
B
C
B
C
A
B
C
A
B
C
A
B
C
D
A
B
B
C
A
B
C
D
A
B
C
D
A
B
C
A
B
C
A
C
D
C
C
A
B
C
D
A
B
C
D
C
D
B
C
B
C
A
B
C
A
B
C
D
C
D
B
C
D
C
B
C
D
B
C
D
C
D
C
D
C
D
E
Lesion length (cm)
D
E
D
E
F
E
F
E
F
F
D
E
F
D
E
F
F
F
F
F
F
D
CRK10Ri-1
CRK10Ri-4
CRK10Ri-13
NH1ox
NH1ox
NH1ox
SFig 5
