# Supplementary material for: A Genetic Screen Identifies a Requirement for Cysteine-Rich–Receptor-Like Kinases in Rice NH1 (OsNPR1)-Mediated Immunity
Source: PLoS Genet. 2016 May 13;12(5):e1006049. doi: 10.1371/journal.pgen.1006049 (PMC4866720; doi:10.1371/journal.pgen.1006049)
Supplement: S8 Fig — Segregating progeny were genotyped for the presence of the GVG-CRK10 transgene. Those containing the transgene are presented in filled bars and the null segregants in open bars. Progeny plants were inoculated with Xoo together with the Kitaake control after DEX induction. Lesion lengths were measured two weeks after inoculation. The results of four lines from one inoculation and another line from another inoculation are presented. Each bar represents the average lesion length and standard deviation of all inoculated leaves from one plant. Progeny of lines #3, #4, #16, and #32 are compared together with the Kitaake control. Progeny of line #21 were compared with its own Kitaake control separately due to the different inoculation time. The letters above each bar show the statistical groupings using the student T-test on each pair based on the 5% significance level within the progeny of each line plus control. (PPT) [file pgen.1006049.s009.ppt]

## Slide 1
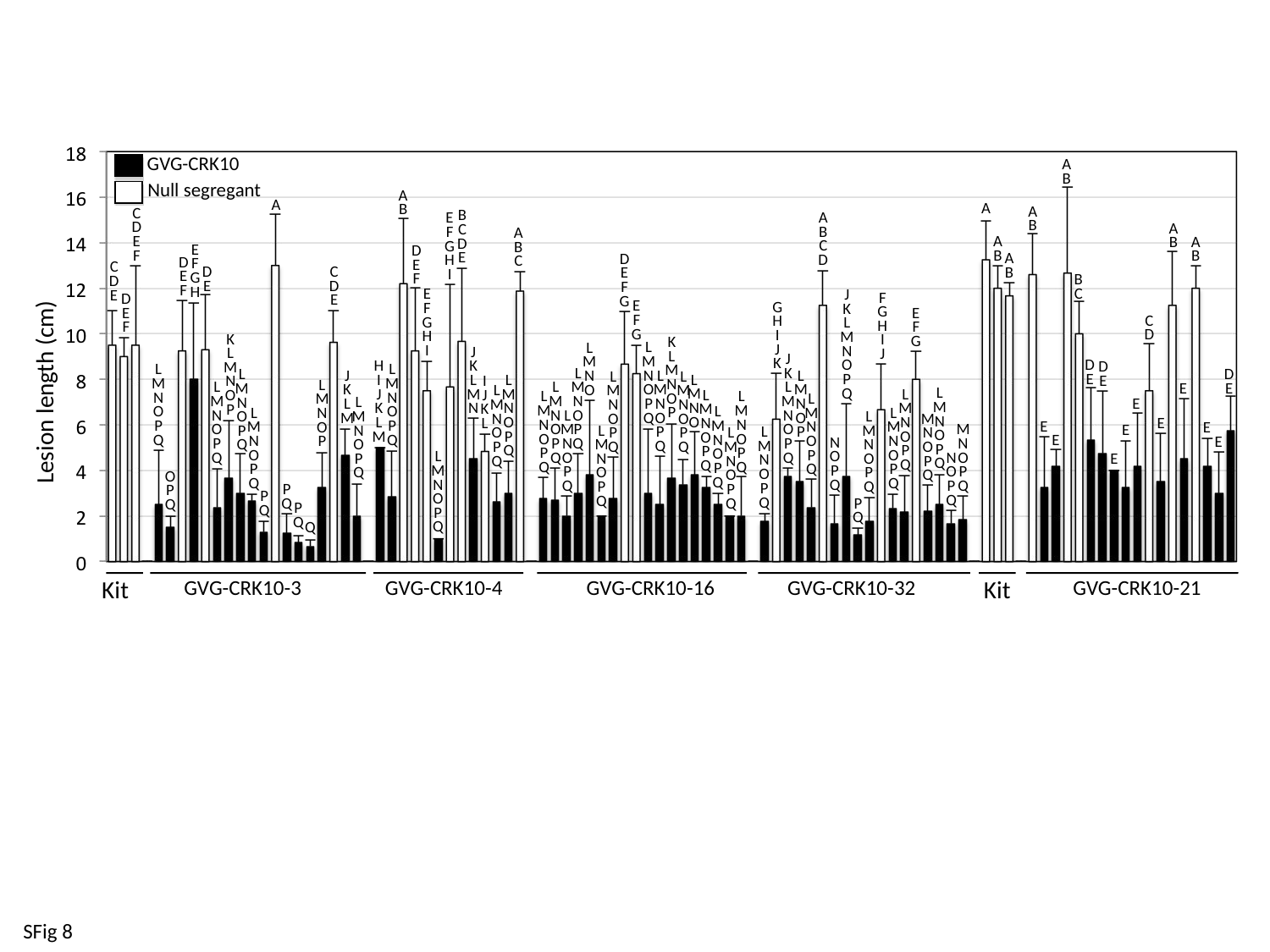

Kit
GVG-CRK10-3
GVG-CRK10-4
GVG-CRK10-16
GVG-CRK10-32
Kit
GVG-CRK10-21
GVG-CRK10
A
B
Null segregant
A
B
A
A
A
B
C
D
E
F
B
C
D
E
A
B
C
D
E
F
G
H
I
A
B
A
B
C
A
B
A
B
E
F
G
H
D
E
F
A
B
D
E
F
G
D
E
F
C
D
E
D
E
C
D
E
B
C
E
F
G
H
I
J
K
L
M
N
O
P
Q
F
G
H
I
J
D
E
F
E
F
G
G
H
I
J
K
E
F
G
C
D
K
L
M
N
O
P
K
L
M
N
O
P
L
M
N
O
P
Q
L
M
N
O
J
K
L
M
N
J
K
L
M
N
O
P
Q
D
E
H
I
J
K
L
M
D
E
L
M
N
O
P
Q
L
M
N
O
P
Q
L
M
N
O
P
Q
L
M
N
O
P
Q
D
E
J
K
L
M
L
M
N
O
P
Q
L
M
N
O
P
L
M
N
O
P
Q
L
M
N
O
P
Q
L
M
N
O
L
M
N
O
P
Q
I
J
K
L
Lesion length (cm)
L
M
N
O
P
L
M
N
O
P
Q
L
M
N
O
P
Q
E
L
M
N
O
P
Q
L
M
N
O
P
Q
L
M
N
O
P
Q
L
M
N
O
P
Q
L
M
N
O
P
Q
L
M
N
O
P
Q
L
M
N
O
P
Q
L
M
N
O
P
Q
E
L
M
N
O
P
Q
L
M
N
O
P
Q
L
M
N
O
P
Q
L
M
N
O
P
Q
L
M
N
O
P
Q
M
N
O
P
Q
E
E
E
M
N
O
P
Q
E
L
M
N
O
P
Q
L
M
N
O
P
Q
L
M
N
O
P
Q
E
E
N
O
P
Q
L
M
N
O
P
Q
N
O
P
Q
E
O
P
Q
P
Q
P
Q
P
Q
P
Q
Q
SFig 8
