# Supplementary material for: A Genetic Screen Identifies a Requirement for Cysteine-Rich–Receptor-Like Kinases in Rice NH1 (OsNPR1)-Mediated Immunity
Source: PLoS Genet. 2016 May 13;12(5):e1006049. doi: 10.1371/journal.pgen.1006049 (PMC4866720; doi:10.1371/journal.pgen.1006049)
Supplement: S9 Fig — CRK6 overexpression transgenic lines carrying the CRK6 gene driven by the maize Ubi-1 promoter were generated in the Kitaake genetic background. (A) T0 plants were inoculated with Xoo and lesion lengths recorded 14 days after inoculation. Fourteen T0 lines are presented. Each bar represents the average and standard deviation of at least 5 leaves. (B) Four CRK6 overexpression lines were tested for CRK6 expression levels compared to the Kitaake control. Each bar represents three replicates. (PPT) [file pgen.1006049.s010.ppt]

## Slide 1
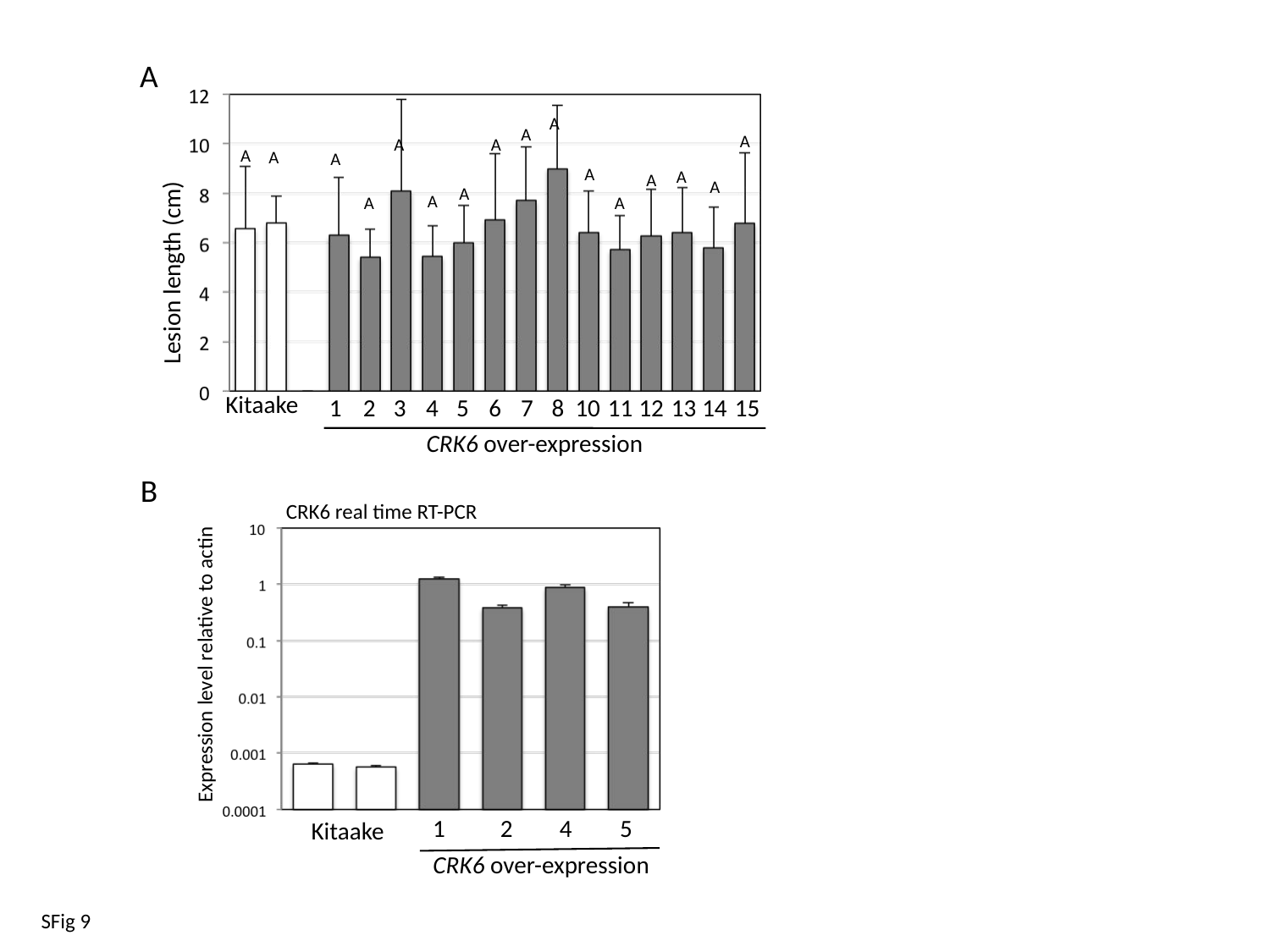

A
A
A
A
A
A
A
A
A
A
A
A
A
A
A
A
A
Lesion length (cm)
Kitaake
1
2
3
4
5
6
7
8
10
11
12
13
14
15
CRK6 over-expression
B
CRK6 real time RT-PCR
Expression level relative to actin
1
2
4
5
Kitaake
CRK6 over-expression
SFig 9
