# Supplementary material for: A Genetic Screen Identifies a Requirement for Cysteine-Rich–Receptor-Like Kinases in Rice NH1 (OsNPR1)-Mediated Immunity
Source: PLoS Genet. 2016 May 13;12(5):e1006049. doi: 10.1371/journal.pgen.1006049 (PMC4866720; doi:10.1371/journal.pgen.1006049)
Supplement: S11 Fig — (A) The CRK6 kinase domain was fused to the His:Nus protein and expressed in E coli BL21 cells. The fusion protein was purified using Ni-NTA resins. A negative control containing a change from aspartate to asparagine at amino acid 488 was also expressed and purified. Kinase activity assay was performed in parallel for the two proteins. (B) The kinase domains of CRK6 and CRK10 are aligned using Geneious to display their similarity. (PPT) [file pgen.1006049.s012.ppt]

## Slide 1
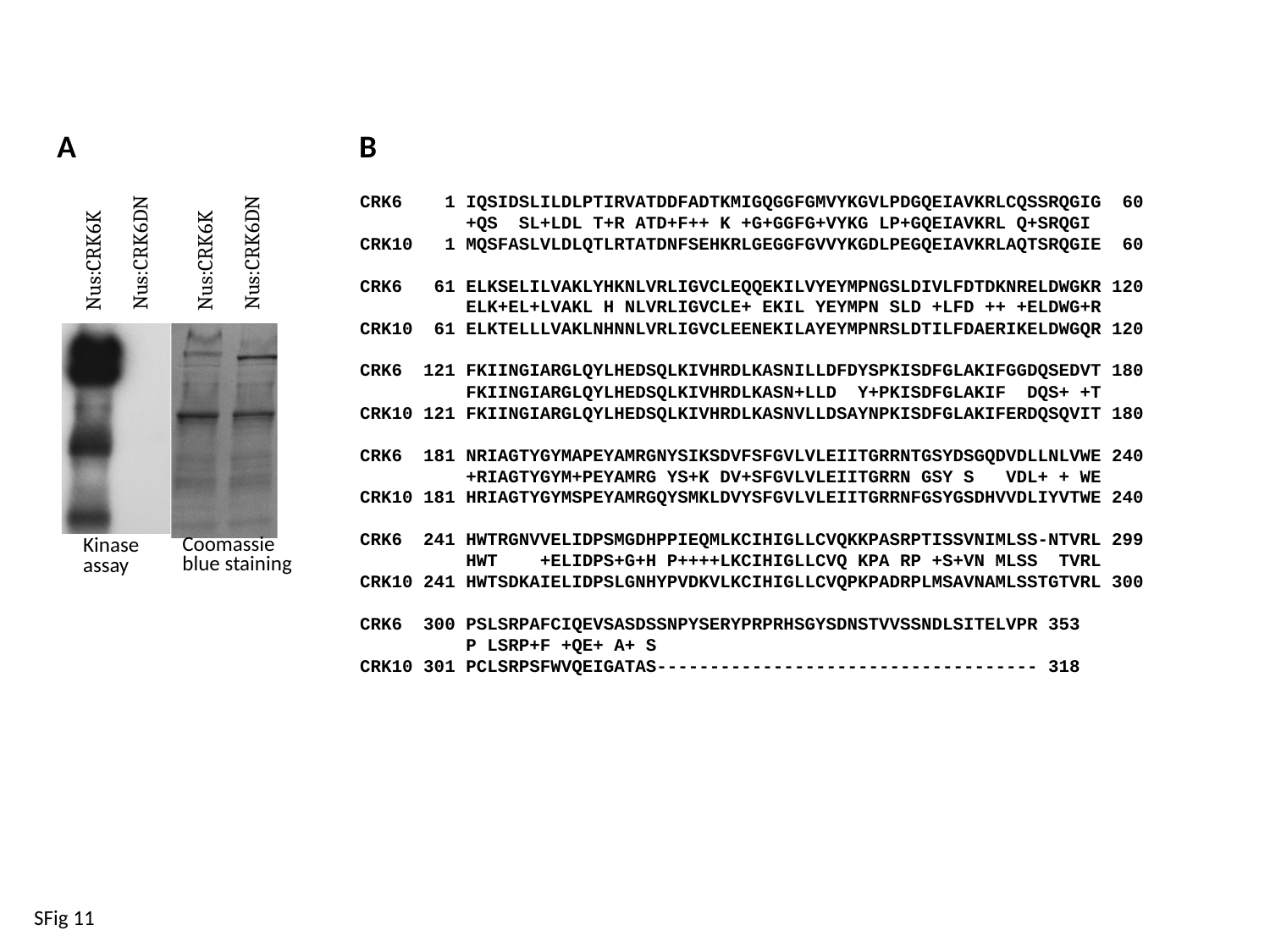

A
B
Nus:CRK6DN
Nus:CRK6DN
Nus:CRK6K
Nus:CRK6K
Coomassie blue staining
Kinase
assay
CRK6    1 IQSIDSLILDLPTIRVATDDFADTKMIGQGGFGMVYKGVLPDGQEIAVKRLCQSSRQGIG  60 
          +QS  SL+LDL T+R ATD+F++ K +G+GGFG+VYKG LP+GQEIAVKRL Q+SRQGI      
CRK10   1 MQSFASLVLDLQTLRTATDNFSEHKRLGEGGFGVVYKGDLPEGQEIAVKRLAQTSRQGIE  60 
CRK6   61 ELKSELILVAKLYHKNLVRLIGVCLEQQEKILVYEYMPNGSLDIVLFDTDKNRELDWGKR 120 
          ELK+EL+LVAKL H NLVRLIGVCLE+ EKIL YEYMPN SLD +LFD ++ +ELDWG+R     
CRK10  61 ELKTELLLVAKLNHNNLVRLIGVCLEENEKILAYEYMPNRSLDTILFDAERIKELDWGQR 120 
CRK6  121 FKIINGIARGLQYLHEDSQLKIVHRDLKASNILLDFDYSPKISDFGLAKIFGGDQSEDVT 180 
          FKIINGIARGLQYLHEDSQLKIVHRDLKASN+LLD  Y+PKISDFGLAKIF  DQS+ +T     
CRK10 121 FKIINGIARGLQYLHEDSQLKIVHRDLKASNVLLDSAYNPKISDFGLAKIFERDQSQVIT 180 
CRK6  181 NRIAGTYGYMAPEYAMRGNYSIKSDVFSFGVLVLEIITGRRNTGSYDSGQDVDLLNLVWE 240 
          +RIAGTYGYM+PEYAMRG YS+K DV+SFGVLVLEIITGRRN GSY S   VDL+ + WE     
CRK10 181 HRIAGTYGYMSPEYAMRGQYSMKLDVYSFGVLVLEIITGRRNFGSYGSDHVVDLIYVTWE 240 
CRK6  241 HWTRGNVVELIDPSMGDHPPIEQMLKCIHIGLLCVQKKPASRPTISSVNIMLSS-NTVRL 299 
          HWT    +ELIDPS+G+H P++++LKCIHIGLLCVQ KPA RP +S+VN MLSS  TVRL     
CRK10 241 HWTSDKAIELIDPSLGNHYPVDKVLKCIHIGLLCVQPKPADRPLMSAVNAMLSSTGTVRL 300 
CRK6  300 PSLSRPAFCIQEVSASDSSNPYSERYPRPRHSGYSDNSTVVSSNDLSITELVPR 353 
          P LSRP+F +QE+ A+ S                                         
CRK10 301 PCLSRPSFWVQEIGATAS------------------------------------ 318
SFig 11
